# Sequencing and Analysis of *Chrysanthemum carinatum* Schousb and *Kalimeris indica.* The Complete Chloroplast Genomes Reveal Two Inversions and *rbcL* as Barcoding of the Vegetable

**DOI:** 10.3390/molecules23061358

**Published:** 2018-06-05

**Authors:** Xia Liu, Boyang Zhou, Hongyuan Yang, Yuan Li, Qian Yang, Yuzhuo Lu, Yu Gao

**Affiliations:** State Key Laboratory of Food Nutrition and Safety, Key Laboratory of Food Nutrition and Safety, Ministry of Education of China, College of Food Engineering and Biotechnology, Tianjin University of Science &Technology, Tianjin 300457, China; zhouboyang456@163.com (B.Z.); yanghongyuan1218@sina.com (H.Y.); 13820282933@163.com (Y.L.); yangqian832402@163.com (Q.Y.); 15822109131@163.com (Y.L.); gaoyu@mail.tust.edu.cn (Y.G.)

**Keywords:** *C. carinatum* Schousb, *K. indica*, chloroplast genome, *Asteraceae*, barcoding, inversion

## Abstract

*Chrysanthemum carinatum* Schousb and *Kalimeris indica* are widely distributed edible vegetables and the sources of the Chinese medicine *Asteraceae*. The complete chloroplast (cp) genome of *Asteraceae* usually occurs in the inversions of two regions. Hence, the cp genome sequences and structures of *Asteraceae* species are crucial for the cp genome genetic diversity and evolutionary studies. Hence, in this paper, we have sequenced and analyzed for the first time the cp genome size of *C. carinatum* Schousb and *K. indica*, which are 149,752 bp and 152,885 bp, with a pair of inverted repeats (IRs) (24,523 bp and 25,003) separated by a large single copy (LSC) region (82,290 bp and 84,610) and a small single copy (SSC) region (18,416 bp and 18,269), respectively. In total, 79 protein-coding genes, 30 distinct transfer RNA (tRNA) genes, four distinct rRNA genes and two pseudogenes were found not only in *C. carinatum* Schousb but also in the *K. indica* cp genome. Fifty-two (52) and fifty-nine (59) repeats, and seventy (70) and ninety (90) simple sequence repeats (SSRs) were found in the *C. carinatum* Schousb and *K. indica* cp genomes, respectively. Codon usage analysis showed that leucine, isoleucine, and serine are the most frequent amino acids and that the UAA stop codon was the significantly favorite stop codon in both cp genomes. The two inversions, the LSC region ranging from *trnC-GCA* to *trnG-UCC* and the whole SSC region were found in both of them. The complete cp genome comparison with other *Asteraceae* species showed that the coding area is more conservative than the non-coding area. The phylogenetic analysis revealed that the *rbcL* gene is a good barcoding marker for identifying different vegetables. These results give an insight into the identification, the barcoding, and the understanding of the evolutionary model of the *Asteraceae* cp genome.

## 1. Introduction

Chloroplasts are crucial for sustaining life on Earth. The chloroplast (cp) genome encodes many key proteins for photosynthesis and other important metabolic processes of plants’ interactions with their environment, such as drought, salt, light, and so on, which give us insights to understand the plant biology, diversity, evolution and climatic adaptation, DNA barcoding and genetic engineering [1,2,3,4,5,6]. Although a relatively conserved architecture and core gene set of cp genomes are shown across higher plants due to the absence of sexual recombination or occurrence of cp capture, considerable variation occurs during evolution [7,8,9,10,11].

A lot of research on the evolution and barcoding of the cp genome in *Asteraceae* was reported [12]. Particularly interestingly, two cp genome regions have a higher inversion frequency in the sunflower family (*Asteraceae*) compared to most eudicots. One region locates the SSC (small single copy) and the other region locates between the *trnC-GCA* to *trnG-UCC* in the LSC (large single copy) region, which maybe help shape our understanding of Compositae evolution and the adaptive versus non-adaptive processes for cellular and genomic complexity [13,14,15,16,17,18,19,20,21,22,23,24,25]. To date, over 2400 sequenced cp genomes (http://www.ncbi.nlm.nih.gov/genomes/) are available. For the *Asteraceae* family, 129 whole cp genomes were sequenced, of which 16 have no inversions in the SSC compared with most other land plants [24,25,26]. However, all 16 species maintain inversion regions in LSC. If there does turn out to be a relationship between inversion and certain evolutionary factors in certain systems, it would depend on the acquisition of more genome sequence information, and the relationship between the cp genome structure/content and the complexity evolutionary factor [27].

The *Asteraceae* (Compositae), or sunflower family is the largest clade in the Asterales and the largest family of flowering plants on Earth. They are angiosperms that appeared about 140 million years ago, comprising 1911 genera and 32,913 species. Nearly one-fourth of all the species of flowering plants belong to the Asteracea, Fabacea, and Orchidaceae families [28,29,30]. The *Asteraceae* family includes a great diversity of species, including annuals, perennials, stem succulents, vines, shrubs, and trees. Commercially important plants in *Asteraceae* include food crops, ornamental plants for their flowers, and species with medicinal properties. *Kalimeris indica* (L.) and *Chrysanthemum carinatum* Schousb both belong to the Asterales family. *Kalimeris indica* (L.) is a member of *Kalimeris* in the sunflower family *Asteraceae* and is variously named as Ma Lan, Ji Er Chang, and Tian Bian Ju in China. *Kalimeris indica* (L.) is not only a traditional Chinese medicine and Miaos’ medicinal plant employed in China to treat colds, diarrhea, gastric ulcers, acute gastric abscess, conjunctivitis, acute orchitis, blood vomiting, and injuries, but it is also a popular vegetable [31,32,33,34,35]. As well as *Kalimeris indica, Chrysanthemum carinatum* Schousb, an important species of *Chrysanthemum* L. in the *Asteraceae* family, is a popular annual herb foodstuff in China, because of its fragrance, flavor, and abundant nutritional value. Moreover, the aerial parts of *Chrysanthemum* L. have been used for the protection or remedy of several diseases in oriental medicinal systems [36,37,38,39,40].

Here, we report the complete cp genome sequence of *Kalimeris indica* (L.) and *Chrysanthemum carinatum* Schousb for the first time. Meanwhile, their gene structure and organization were analyzed. Furthermore, the whole cp genome sequences were compared with other genii of the *Asteraceae* family, especially the inversions, which were found and compared. Phylogenetic analyses of DNA sequences for *rcbL* of 24 plant species indicated that *rcbL* would be a good molecular barcoding target.

## 2. Results

### 2.1. Features of the C. carinatum Schousb and K. indica cp Genome

**Figure 1:**
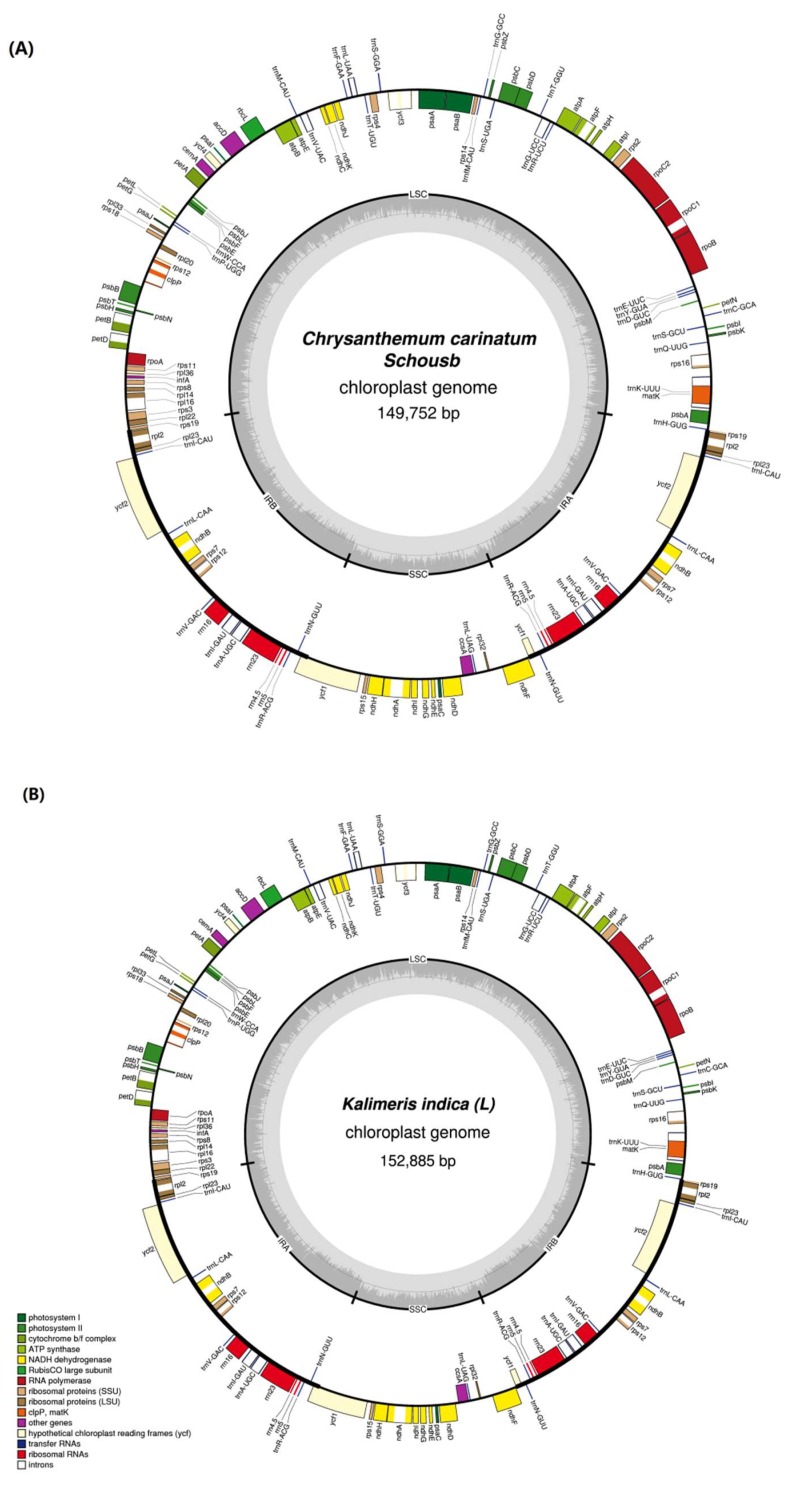
The complete cp genome map of *C. carinatum Schousb* and *K. indica*. (**A**) *C. carinatum Schousb*; (**B**) *K. indica*. The genes marked inside the circle are transcribed clockwise, and those outside are counterclockwise. Genes are color-coded according to their function.

The complete cp genome of *C. carinatum* Schousb and *K. indica* have a typical quadripartite structure and are 149,752 bp and 152,885 bp in size, respectively (Figure 1). *C. carinatum* Schousb has an LSC region of 82,290 bp and an SSC region of 18,416 bp which are separated by a pair of IR regions of 24,523 bp (Table 1 and Figure 1A). *K. indica* has an LSC region of 84,610 bp ranging from *trnH-GUG* to *rps19* (Ribosomal protein S19), a SSC region of 18,269 bp from *ycf1* (hypothetical protein 1 gene) to *ndhF* (NAD(P)H dehydrogenase), a pair of IR regions of 25,003 bp from *rps19* to *ycf1* and ranging from pseudogene *ycf1* to pseudogene *rps19*, respectively (Table 1 and Figure 1B). Both of them have two inversions like most of the sunflower family species, one inversion occurs in the whole SSC region and the other inversion region is located in the LSC from trnC-GCA to trnG-UCC [13,15,41].

From Table 1, the overall GC content of the *C. carinatum* Schousb and *K. indica* cp genome is the same (37.5%), with a higher GC content (43.1% and 43%) in the IR regions than in the LSC (35.7% and 35.2%) and SSC regions (30.8% and 31.2%), which is similar to other *Asteraceaes* [11,12,13,14,15,17,18,25,26,42]. 

The AT content in the third, second and first codon position of the *C. carinatum* Schousb cp genome are 70.4%, 61.9%, and 54.3%, and the AT content in the third, second, and first codon position of *K. indica* are 69.8%, 61.9%, and 54.5% (Table 1). The results showed that the third codon position and the second codon position have significantly higher AT representation, which is a common feature of the cp genome [43,44,45,46].

### 2.2. Functional Genes of the C. carinatum Schousb and K. indica cp Genome

There are 113 unique functional genes and two pseudogenes in the *C. carinatum* Schousb or *K. indica* cp genome (Table 2). Among the 113 functional genes, 79 protein-coding genes, 30 distinct transfer RNA (tRNA) genes, and four distinct rRNA genes were found not only in the *C. carinatum* Schousb genome but also in the *K. indica* cp genome (Table 2). Notably, seven protein-coding, seven tRNA, and all the rRNA genes are duplicated in the IR regions, which is common in most cp genomes [47,48]. Meanwhile, the coding regions constitute 51.6% and 51.3% of the genome in the *C. carinatum* Schousb and *K. indica* cp genome, respectively. The non-coding regions constitute 48.4% and 48.7% including the introns, pseudogenes, and intergenic spacers, respectively. Two pseudogenes (*ycf1* and *rps19*) are both found in both *C. carinatum* Schousb and *K. indica* cp genomes, a common feature shared with cp genomes from other plants [25,47,48].

Among the *C. carinatum* Schousb or *K. indica* cp genomes, a total of 18 genes (six tRNA genes and 12 protein-coding genes) contain introns (Table 3). They are mostly located in the LSC region (13 genes), with only one located in the SSC region, and four genes locate in the IR regions. Like other angiosperms, *clpP*, *rps12,* and *ycf3* contain two introns [48]. Interestingly, *rps12* was found to be a trans-spliced gene, whose 5′ end is located in the LSC region and the duplicated 3′ end is located in the IR region. Consistent with many research results, the largest intron is in the *trnK-UUU* gene, which contains the *matk* gene in the intron. Moreover, *trnL-UAA* has the smallest intron in both of them. Comparing these 18 introns between *C. carinatum* Schousb and *K. indica*, most of them are in *K. indica* and are longer than those in *C. carinatum* Schousb, whereas the introns of *rpl16, trnK-UUU,* and the introII *rps12* are a bit shorter, and the *trnV-UAC* intron is the same size, which may be one reason why the whole cp genome of *K. indica* is larger than *C. carinatum* Schousb.

### 2.3. Codon Usage of the K. indica and C. carinatum Schousb cp Genome

As shown in Table 4 and Table 5, a total of 25,415 and 26,124 codons are involved in the protein-coding in *C. carinatum* Schousb and *K. indica*, respectively. Of these codons, leucine, isoleucine, and serine are the most frequent amino acids in *C. carinatum* Schousb, which encode in 2778 (10.93%), 2195 (8.64%), and 2080 (8.18%) codons, respectively. Leucine, isoleucine, and serine are the most frequent amino acids in *K. indica*, which encode in 2795 (10.70%), 2190 (8.38%) and 2131 (8.16%) codons, respectively. Both of them contain 85 stop codons, which is the least frequent codon. The cysteine codons are the least frequent universal amino acid, which has 286 (1.13%) and 294 (1.13%) codons in *C. carinatum* Schousb and *K. indica*, respectively.

Usually, relative synonymous codon usage (RSCU) can be divided into four types, including lack of bias (RSCU < 1.0), low bias (1.0 < RSCU< 1.2), moderately biased (1.2 < RSCU< 1.3) and highly biased (RSCU > 1.3) [49,50]. As shown in Table 4 and Table 5, it is uncannily similar that there are 32 lack of bias codons, two no-bias codons with RSCU = 1 (tryptophan and methionine), and 21 highly biased codons, with the exception of five low bias codons in *C. carinatum* Schousb but two low bias codons in *K. indica*, and three moderately biased codons in *C. carinatum* Schousb but six moderately biased codons in *K. indica,* respectively. The UAA stop codon was the significantly favorite stop codon in the cp genomes. The results showed that the RSCU was significantly biased except for tryptophan and methionine in *C. carinatum* Schousb and *K. indica* and that the A/T ending is very rich, which is popular in the cp genomes of higher plants [51,52].

### 2.4. Repeats Structure and SSRs

**Figure 2:**
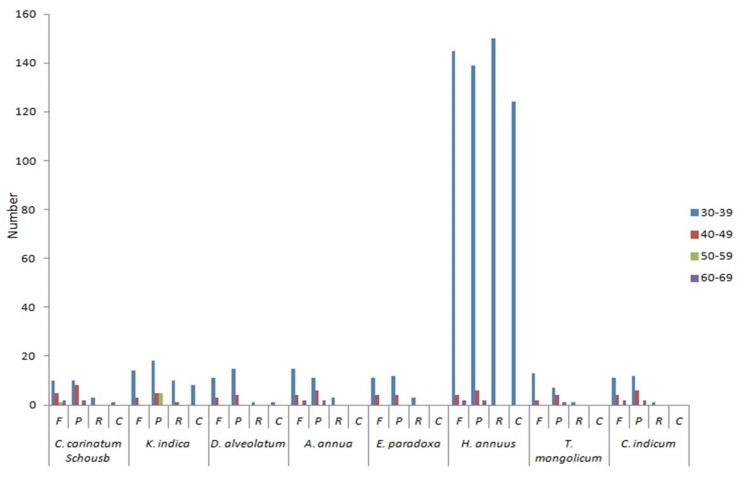
The repeat sequences of eight *Asteraceae* cp genomes. *F* (forward), *P* (palindrome), *R* (reverse), and *C* (complement) represent the repeat types. Different colours represent the repeats in different lengths.

Analysis of the repeat structure showed that the total of 42 repeats of *C. carinatum* Schousb is significantly less than *K. indica* (59), which may be one reason for the larger cp genome of *K. indica*. There are 18 forward repeats, 20 palindromic repeats, three reverse repeats and one complement repeats in the cp genome of the *C. carinatum* Schousb (Table 6) and 17 forward repeats, 28 palindromic repeats, 11 reverse repeats, and eight complement repeats in the *K. indica* cp genome (Table 7). All of them range from 30 to 60 bp in length and are mostly located in the intergenic spacer (IGS) and intron sequences. Comparison analysis of the repeats of six other species of *Asteraceae* showed that *H. annuus* contained the most repeats (572) and *T. mongolicum* contained the fewest repetitive sequences (28) (Figure 2). Among the eight species of *Asteraceae*, we also found that the base fragment with the most repeats was between 30–39 bp in length. Complement repeats are relatively richer than in other families, although *A. annua*, *E. paradoxa, T. mongolicum* and *C. indicum* do not contain complement repeats [48].

There are 70 and 90 simple sequence repeats (SSRs) in the cp genome of *C. carinatum* Schousb and *K. indica,* respectively (Table 8 and Table 9). Most of them are mononuclear repeats; 43 in *C. carinatum* Schousb and 30 in *K. indica,* respectively. Eight dinucleotide repeats, five trinucleotide repeats, thirteen tetranucleotide repeats, and one pentanucleotide repeats were also found in the *C. carinatum* Schousb cp genome. Eighteen dinucleotide repeats, twenty-eight trinucleotide repeats, seven tetranucleotide repeats, and seven pentanucleotide repeats were also discovered in *K. indica*. The majority of SSRs are located in the LSC region, 81.43% (*C. carinatum* Schousb) and 78.89% (*K. indica*) of SSRs are located in the LSC (Table 8 and Table 9) whereas, only twelve (*C. carinatum* Schousb) and 9 (*K. indica*) SSRs are located in the CDSs (Table 10). Comparing with the six other species of *Asteraceae*, the most SSRs and the least SSRs located in CDS were found in the *K. indica* cp genome. Furthermore, most of the SSRs are AT repeats, which is consistent with the AT richness [48,51].

### 2.5. Comparison of the Whole cp Genome of Asteraceae

**Figure 3:**
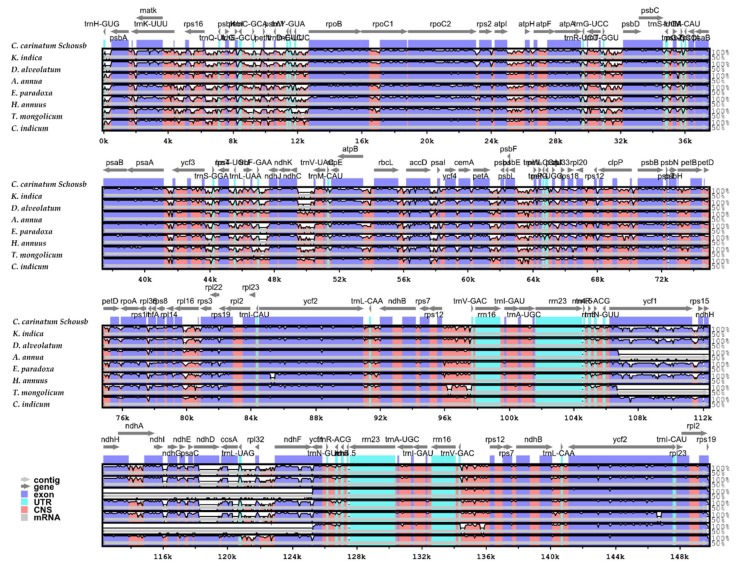
The comparison of eight *Asteraceae* cp genomes by using mVISTA. The grey arrows above the contrast indicate the direction of the gene translation.The y-axis represents the percent identity between 50% and 100%. Protein codes (exon), rRNA, tRNA and conserved non-coding sequence (CNS) are shown in different colors, respectively.

Using *C. carinatum* Schousb as a reference, mVISTA was used to compare the overall sequence of the cp genome of the eight *Asteraceae* species (Figure 3). It was found that the cp genome sequence of *C. carinatum* Schousb had the shortest size, whereas the *K. indica* cp genome had the longest size among the eight *Astareace* species. The difference of the sequence length was mainly due to the difference of length of the LSC region and there was no significant differences in the SSC and IRs regions’ length. It is important to note that the SSC regions of *A. annua* and *T. mongolicum* are completely different from *C. carinatum* Schousb because of the inversion in most of the *Asteraceae* species, whereas the inversion region in LSC compared to other eudicots is relatively constant [13,14,15,16,17,18,19,20,21,22,23,24,25]. In order to illustrate this, we compared the cp genomes between 129 species of *Asteraceae* and some cp genomes of non-*Asteraceae* species using the mVISTA software to confirm the structural changes (Data not shown). Among them, only 16 species of *Asteraceae* (*Artemisia argyi, Artemisia capillaries, Artemisia frigida, Artemisia gmelinii, Artemisia montana, Aster altaicus, Carthamus tinctorius, Centaurea diffusa, Eclipta prostrate, Lactuca sativa, Saussurea chabyoungsanica, Saussurea involucrate, Saussurea polylepis, Taraxacum mongolicum, Taraxacum officinale, Taraxacum platycarpum*) did not invert in the SSC region (Supplemental Table S1). However, the LSC region was generally inverted. In addition, the coding area is more conservative than the non-coding area [53,54].

### 2.6. IR Contraction and Expansion

**Figure 4:**
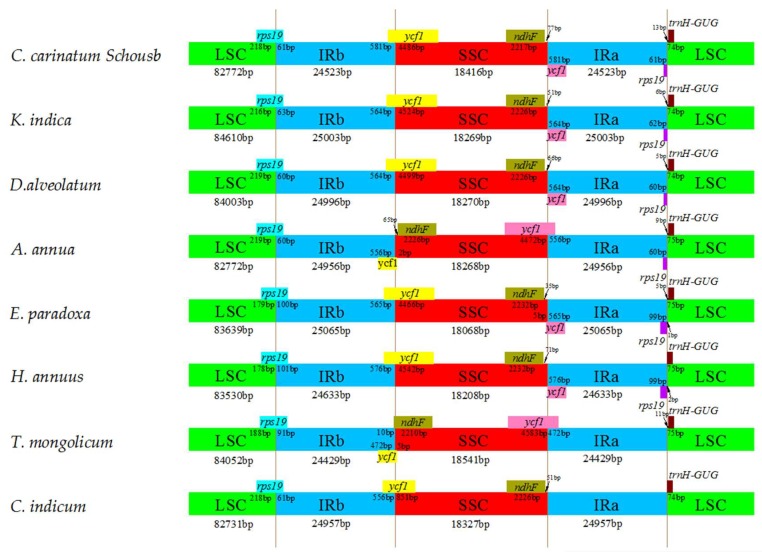
The comparison of the borders of the LSC, SSC, and IR regions among the eight *Asteraceae* cp genomes.

The IR contraction and expansion of *C. carinatum* Schousb and *K. indica* were analyzed by comparing the LSC/IRB/SSC/IRA boundary among the eight species of *Asteraceae* (Figure 4). For all of them, the junction of the IRB and LSC regions was connected by the *rps19* gene such that pseudogenes *rps19* in the IRA/LSC boundaries were usually found in the duplication of the 3′end of the *rps19* in IRA, in which the pseudogene *rps19* lengths ranged from 60 to 99 bp. The *ycf1* gene was located in both IRB/SSC and IRA/LSC boundaries. Additionally, 477 to 581 bp pseudogenes *ycf1* at the border of IRA/SSC were produced. Among these eight cp genomes, *C. carinatum* Schousb had the largest *ycf1* pseudogene (581 bp in length), whereas the inversion of the SSC region in *A. annua* and *T. mongolicum* caused the pseudogene *ycf1* to appear at the border of IRB/SSC. *T. mongolicum* and *C. indicum* showed the absence of the *ycf1* pseudogene and the *rps19* pseudogene. With the exception of *T. mongolicum*, all *ndhF* genes were located in the SSC region, which has a distance between 35–77 bp from the IRA border, and the *ndhF* gene of *C. carinatum* Schousb has the largest distance to IRA border. Furthermore, the *ndhF* gene of *T. mongolicum* is 10 bp across the IRB region and intersects with the pseudogene *ycf1*. The *trnH-GUG* genes are located in the LSC region between 0–13 bp from the IRA border and *C. carinatum* Schousb has the largest one. Oddly, a relatively smaller IR size but longer pseudogene *ycf1* length were found in *C. carinatum* Schousb, which may be due to the occurrence of the contraction in the intergenic regions in *C. carinatum* Schousb [48].

### 2.7. Phylogenetic Analysis and Barcoding

**Figure 5:**
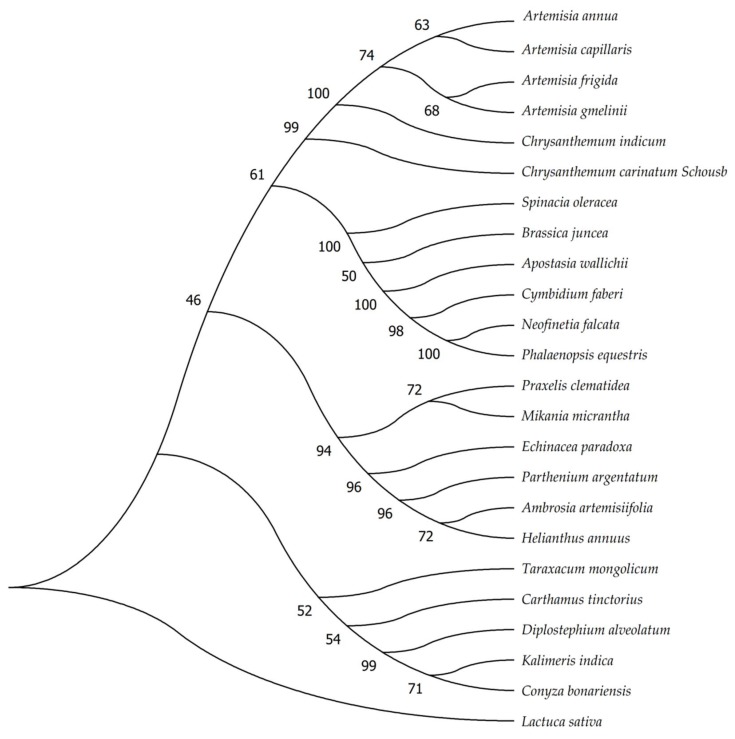
The molecular phylogenetic analysis of the cp protein-coding gene *rcbL* for 24 samples using the Maximum Likelihood method. The tree was constructed by using MEGA7. The stability of each tree node was tested by bootstrap analysis with 1000 replicates.

The cp genome is a useful resource and tool for taxonomy, determining evolutionary relationships within families, and DNA barcoding [42,55,56,57,58,59]. Here, for obtaining a useful barcoding marker and a reasonable phylogenetic status of *C. carinatum* Schousb and *K. indica*, we established the phylogenetic tree of 24 sample species using the Maximum Likelihood (ML) method by the alignment of 18 genes (*atpF, clpP, matK, ndhA, ndhB, ndhF, petB, petD, psaB, psbA, rbcL, rpl2, rpl16, rpoB, rpoC1, rps12, rps16, rps19*), respectively (Supplemental Figure S1). These 24 species include 18 *Asteraceae* species, four *Orchidaceae* species, one *Chenopodiaceae* species and one Cruciferae species. We compared six non-*Asteraceae* species with 18 *Asteraceae* species because the former are morphologically similar to *C. carinatum* Schousb and *K. indica*. From the results of the alignment, the *matK*, *ndhF* and *rbcL* genes are better than the others. There are eight nodes that had bootstrap values >90% when *matK* and *ndhF* were used for gene alignment. However, they both had three nodes with bootstrap values <40% and one node even had a bootstrap value <30%. As for the *rbcL* gene, 10 out of the 19 nodes had bootstrap values >90%. The minimum bootstrap value is 46% and the rest of the nodes had bootstrap values >50%. We show the phylogenetic tree by *rbcL* gene alignment in Figure 5, which shows that *C. carinatum* Schousb is a closely related species with *Chrysanthemum indicum* and that *K. indica* is closely related with the *Conyza bonariensisspecies,* which is consistent with their classification and morphology. Hence, the *rbcL* gene could be a good candidate gene to be used as a barcoding marker [51,52,54].

## 3. Discussion

We reported on the two genome sequence of *C. carinatum* Schousb and *K. indica*, which provides an important resource to study the evolutionary and inversion mechanism as well as the molecular barcoding of vegetables in the *Asteraceae* family. Although cp genomes of Angiosperms are well-conserved in the genomic structure, the inversion and IR expansion contraction occur frequently [7,8,9,10,11]. These results showed that the inversion of *trnC-GCA* to *trnG-UCC* in the LSC and the whole SSC occurred in these two species, which are congruent with most of the cp genomes of *Asteraceae* family [41,60,61,62]. Oddly, though some no-inversion occurs in the SSC region, the inversion of *trnC-GCA* to *trnG-UCC* in the LSC does not occur, which maybe could make amplification of *trnC-GCA* or *trnG-UCC* boundary regions a good resource for the molecular taxonomy of *Asteraceae.* Meanwhile, the SSC/IRA and LSC/IRB border extends into the *ycf1* and *rps19* with the subsequent formation of the *ycf1* and *rps19* pseudogenes, respectively [63,64]. The *ycf1* and *rps19* pseudogenes occur in most of the cp genomes, with deletion regions of similar size in species of the same family, but different deletion size between different families (Figure 4) [48].

Here, the analysis of the codon usage frequency and RSCU showed that leucine and isoleucine are the most popular amino acids in the cp genomes in the *C. carinatum* Schousb and *K. indica* as Angiosperms [23,52,65,66,67]. Meanwhile, a significantly higher T bases appearance was indicated in the 3th CDS position than in the 2nd position or 1st position. The T bases appear at the end of most favorite synonymous codons (RSCU > 1.0), but the reverse occurs for the G base (Table 1, Table 4 and Table 5). Consistent with most earlier reports about repeats and SSRs, the mononucleotide repeats with A/T repeats are more abundant, which may lead to AT richness in the Angiosperm cp genomes [68,69,70].

Here, we compared the whole cp genome of six species of *Asteraceae*, which revealed that the inversion is usual in most *Asteraceae* species, with some no-inversion occuring only in the SSC regions’ inversions, but not in the inversions of *trnC-GCA* to *trnG-UCC* in the LSC. Among the 129 *Asteraceae* species that have been sequenced, inversion occurs in the LSC region. Whether or not the SSC region is inverted is closely related to the genus. For example, *Diplostephium* has the most sequenced species and all of them have inverted SSC regions. Inconsistent inversions within the genus only occurred in the *Aster* genus and the *Taraxacum* genus (Supplemental Table S1). This phenomenon may be a key feature of the cp genome of *Asteraceae*. It provides an insight into future evolution research.

Here, the *rbcL* genes represents a good diversity among some vegetables. The closer the relationship between species, the higher the sequence similarity of the genes. The phylogenetic tree of the *rbcL* gene among the *Asteraceae* species can obtain a better discrimination result. More importantly, it also could differentiate different family species, which illustrate that the *rbcL* gene is not only highly distinguished within the *Asteraceae* family, but also highly distinguished within most of vegetables. So, *rbcL* would be one of the best choices for DNA barcoding to distinguish between vegetables [71,72,73].

## 4. Materials and Methods

### 4.1. DNA Extraction and Sequencing

The total DNA was extracted from about 100 g fresh leaves of *C. carinatum* Schousb and *K. indica* using the CTAB method [74]. The total DNA quantity was evaluated by the value of the ratio of absorbance measurements at 260 nm and 280 nm (A260/A280) using Nanodrop2000 (Thermo Fisher Scientific, Waltham, MA, USA), whereas visual assessment of the DNA size and integrity was performed using gel electrophoresis. The DNA was sheared to fragments of 300~500 bp. Paired-end libraries were prepared wih the TruSeq^TM^ DNA Sample Prep Kit and the TruSeq PE Cluster Kit. The genome was then sequenced using the HiSeq4000 platform (Illumina, Santiago, CA, USA).

### 4.2. Genome Assembly

The assembly of the cp genome of *C. carinatum* Schousb and *K. indica* were first carried out through the error correction and production of initial contigs by the GS FLX De Novo Assembler Software (Newbler V2.6). PCR amplification and Sanger sequencing were performed to verify the four-f- junction regions between the IRs and the LSC/SSC. The final cp genome of *C. carinatum* Schousb and *K. indica* were submitted to the Genbank with the accession number MG710386 and MG710387, respectively.

### 4.3. Gene Annotation and Codon Usage Analysis

The cp genome was annotated using BLAST [75] and DOGMA with manual corrections [76]. The tRNAscan-SE was used to identify the tRNA genes [77]. OGDRAW was used to draw the circular genome map [78]. MEGA5 was used to analyze the characteristics of the variations in the synonymous codon [79]. The relative synonymous codon usage values (RSCU), codon usage, and GC content were also determined by MEGA7 [80].

### 4.4. Repeat Structure and Single Sequence Repeats (SSRs) Analysis

Analysis of tandem repeats with more than 30 bp and a minimum of 90% sequence (forward, palindromic, reverse, and complement) and single sequence repeats (SSRs) was performed by REPuter [81] and MISA [82], respectively, with the same parameters as described in Ni et al. [53].

### 4.5. Comparative Genome Analysis of the C. carinatum Schousb and K. indica Genomes

Comparison of the overall cp genome of *C. carinatum* Schousb and *K. indica* with six cp genomes of *Asteraceae* was performed by mVISTA [83,84] using the annotation of *C. carinatum* Schousb as a reference.

### 4.6. Phylogenetic Analysis

A total of 24 complete cp genome sequences were downloaded from the NCBI Organelle Genome Resources database. MEGA7 [80] was used to construct the evolutionary tree of the cp protein-coding gene *rcbL* of 24 samples by the Maximum Likelihood method.

## 5. Conclusions

In this study, the complete cp genome of *C. carinatum* Schousb and *K. indica* were reported and analyzed for the first time. Both of them are key traditional Chinese medicines and edible vegetables. Comparing them with other *Asteraceae* species, the cp genome of these two species display two inversions, one is *trnC-GCA* to *trnG-UCC* in the LSC and other is the whole SSC region. We found that most of the inversions of *trnC-GCA* to *trnG-UCC* almost happened in every cp genome of the *Asteraceae* species. Seventy and ninety simple sequence repeats (SSRs) were present in the cp genome of *C. carinatum* Schousb and *K. indica*, respectively. Certainly, these results provide good chances for developing barcoding molecular markers for different families distinguish, which can be obtained through combination by different barcoding markers or through the boundary region marker. The *rbcL* is a good barcoding marker. Meanwhile, these results would be useful for the evolutionary study of *C. carinatum* Schousb and *K. indica*, and might also contribute to the genetics and barcoding of easily-confused leafy vegetables.

## Figures and Tables

**Table 1:** The base composition in the *C. carinatum Schousb* and *K. indica* cp genomes.

Region	*C. carinatum Schousb*					*K. indica*				
	T(U) (%)	C (%)	A (%)	G (%)	Length (bp)	T(U) (%)	C (%)	A (%)	G (%)	Length (bp)
LSC	32.4	17.6	32.0	18.1	82,290	32.6	17.3	32.2	17.9	84,610
SSC	35.1	14.7	34.1	16.1	18,416	34.9	14.8	33.9	16.4	18,269
IRa	28.3	22.3	28.6	20.8	24,523	28.3	22.2	28.7	20.8	25,003
IRb	28.6	20.8	28.3	22.3	24,523	28.7	20.8	28.3	22.2	25,003
Total	31.8	19.0	30.7	18.5	149,752	31.8	19.0	30.7	18.5	152,885
CDS	31.5	17.7	30.7	20.1	77,289	31.5	17.7	30.6	20.3	78,372
1st position	23.7	18.9	30.6	26.7	25,763	23.9	18.8	30.6	26.8	26,124
2nd position	32.6	20.4	29.3	17.7	25,763	32.6	20.3	29.3	17.8	26,124
3rd position	38.3	13.7	32.1	15.9	25,763	38.0	13.9	31.8	16.3	26,124

**Table 2:** The genes present in the *C. carinatum* Schousb and *K. indica* cp genomes.

Group of Genes	*C. carinatum* Schousb Gene Names	*K. indica* Gene Names
Photosystem I	*psaA, psaB, psaC, psaI, psaJ*	*psaA, psaB, psaC, psaI, psaJ*
Photosystem II	*psbA, psbB, psbC, psbD, psbE, psbF, psbH, psbI, psbJ, psbK, psbL, psbM, psbN, psbT, psbZ*	*psbA, psbB, psbC, psbD, psbE, psbF, psbH, psbI, psbJ, psbK, psbL, psbM, psbN, psbT, psbZ*
Cytochrome b/f complex	petA, petB *, petD *, petG, petL, petN	petA, petB *, petD *, petG, petL, petN
ATP synthase	atpA, atpB, atpE, atpF *, atpH, atpI	atpA, atpB, atpE, atpF *, atpH, atpI
NADH dehydrogenase	ndhA *, ndhB * (×2), *ndhC, ndhD, ndhE, ndhF, ndhG, ndhH, ndhI, ndhJ, ndhK*	ndhA *, ndhB * (×2), ndhC, ndhD, ndhE, ndhF, ndhG, ndhH, ndhI, ndhJ, ndhK
RuBisCO large subunit	*rbcL*	*rbcL*
RNA polymerase	rpoA, rpoB, rpoC1 *, rpoC2	rpoA, rpoB, rpoC1 *, rpoC2
Ribosomal proteins (SSU)	rps2, rps3, rps4, rps7 (×2), rps8, rps11, rps12 ** (×2), rps14, rps15, rps16 *, rps18, rps19	*rps2, rps3, rps4, rps7* (×2), rps8, rps11, rps12 ** (×2), rps14, rps15, rps16 *, rps18, rps19
Ribosomal proteins (LSU)	rpl2 * (×2), rpl14, rpl16 *, rpl20, rpl22, rpl23 (×2), *rpl32, rpl33, rpl36*	rpl2 * (×2), rpl14, rpl16 *, rpl20, rpl22, rpl23 (×2), *rpl32, rpl33, rpl36*
Miscellaneous proteins	accD, clpP **, matK, ccsA, cemA, infA	accD, clpP **, matK, ccsA, cemA, infA
Hypothetical chloroplast reading frames (ycf)	*ycf1, ycf2* (×2), ycf3 **, ycf4	*ycf1, ycf2* (×2), ycf3 **, ycf4
Transfer RNAs	*trnA-UGC* (×2)*, trnC-GCA, trnD-GUC, trnE-UUC, trnF-GAA, trnG-GCC, trnG-UCC, trnH-GUG, trnI-CAU* (×2), *trnI-GAU* (×2), *trnK-UUU, trnL-CAA*(×2)*, trnL-UAA, trnL-UAG, trnfM-CAU, trnM-CAU, trnN-GUU* (×2), *trnP-UGG, trnQ-UUG, trnR-ACG* (×2)*, trnR-UCU, trnS-GCU, trnS-GGA, trnS-UGA, trnT-GGU, trnT-UGU, trnV-GAC* (×2)*, trnV-UAC, trnW-CCA, trnY-GUA*	*trnA-UGC* (×2)*, trnC-GCA, trnD-GUC, trnE-UUC, trnF-GAA, trnG-GCC, trnG-UCC, trnH-GUG, trnI-CAU* (×2), *trnI-GAU* (×2), *trnK-UUU, trnL-CAA*(×2)*, trnL-UAA, trnL-UAG, trnfM-CAU, trnM-CAU, trnN-GUU* (×2), *trnP-UGG, trnQ-UUG, trnR-ACG* (×2)*, trnR-UCU, trnS-GCU, trnS-GGA, trnS-UGA, trnT-GGU, trnT-UGU, trnV-GAC* (×2)*, trnV-UAC, trnW-CCA, trnY-GUA*
Ribosomal RNAs	*rrn4.5* (×2), *rrn5* (×2), *rrn16 (×2), rrn23* (×2)	*rrn4.5* (×2), *rrn5* (×2), *rrn16* (×2), *rrn23* (×2)
Pseudogenes	*ycf1, rps19*	*ycf1, rps19*
Total	132	132

(×2) indicates a duplicated gene; * represents the introns that the gene contains; ** indicates there are two introns that the gene contains.

**Table 3:** The intron-containing genes of the *C. carinatum Schousb* and *K. indica* cp genomes and the lengths of the introns and exons.

*C.* Gene	Location	Exon I (bp)	Intron I (bp)	Exon II (bp)	Intron II (bp)	Exon III (bp)	*K.* Gene	Location	Exon I (bp)	Intron I (bp)	Exon II (bp)	Intron II (bp)	Exon III (bp)
*atpF*	LSC	145	699	410			*atpF*	LSC	145	709	410		
*clpP*	LSC	71	796	291	611	229	*clpP*	LSC	71	814	291	615	229
*ndhA*	SSC	539	1045	553			*ndhA*	SSC	553	1064	539		
*ndhB*	IR	777	670	756			*ndhB*	IR	777	674	756		
*petB*	LSC	6	751	642			*petB*	LSC	6	823	642		
*petD*	LSC	8	675	475			*petD*	LSC	8	809	475		
*rpl16*	LSC	9	1029	399			*rpl16*	LSC	9	1098	399		
*rpl2*	IR	391	664	434			*rpl2*	IR	391	671	434		
*rpoC1*	LSC	432	733	1641			*rpoC1*	LSC	432	742	1638		
rps12 *	LSC	114	-	232	536	26	rps12 *	LSC	114		232	535	26
*rps16*	LSC	41	891	184			*rps16*	LSC	41	876	226		
*trnA-UGC*	IR	38	812	35			*trnA-UGC*	IR	35	820	38		
*trnG-UCC*	LSC	23	722	47			*trnG-UCC*	LSC	23	732	48		
*trnI-GAU*	IR	43	775	35			*trnI-GAU*	IR	38	780	35		
*trnK-UUU*	LSC	37	2562	30			*trnK-UUU*	LSC	37	2539	35		
*trnL-UAA*	LSC	37	422	50			*trnL-UAA*	LSC	37	438	50		
*trnV-UAC*	LSC	38	573	37			*trnV-UAC*	LSC	38	573	37		
*ycf3*	LSC	125	698	229	735	153	*ycf3*	LSC	125	702	229	739	153

* The *rps12* gene is a trans-spliced gene with the 5′ end located in the LSC region and a duplicated 3′ end in the IR region.

**Table 4:** The codon-anticodon recognition pattern and codon usage for the *C. carinatum* Schousb cp genome.

Amino Acid	Codon	No.	RSCU	tRNA	Amino Acid	Codon	No.	RSCU	tRNA
Phe	UUU	956	**1.32**		Stop	UAA	49	**1.73**	
Phe	UUC	496	0.68	*trnF-GAA*	Stop	UAG	21	0.74	
Leu	UUA	874	**1.89**	*trnL-UAA*	Stop	UGA	15	0.53	
Leu	UUG	553	**1.19**	*trnL-CAA*	His	CAU	452	**1.52**	
Leu	CUU	620	**1.34**		His	CAC	143	0.48	*trnH-GUG*
Leu	CUC	183	0.40		Gln	CAA	703	**1.51**	*trnQ-UUG*
Leu	CUA	358	0.77	*trnL-UAG*	Gln	CAG	227	0.49	
Leu	CUG	190	0.41		Asn	AAU	978	**1.56**	
Ile	AUU	1075	**1.47**		Asn	AAC	277	0.44	*trnN-GUU*
Ile	AUC	428	0.58	*trnI-GAU*	Lys	AAA	1026	**1.50**	*trnK-UUU*
Ile	AUA	692	0.95	*trnI-CAU*	Lys	AAG	345	0.50	
Met	AUG	613	1.00	*trn(f)M-CAU*	Asp	GAU	831	**1.59**	
Val	GUU	490	**1.44**		Asp	GAC	214	0.41	*trnD-GUC*
Val	GUC	166	0.49	*trnV-GAC*	Glu	GAA	986	**1.50**	*trnE-UUC*
Val	GUA	523	**1.54**	*trnV-UAC*	Glu	GAG	331	0.50	
Val	GUG	178	0.52		Cys	UGU	198	**1.38**	
Ser	UCU	580	**1.77**		Cys	UGC	88	0.62	*trnC-GCA*
Ser	UCC	313	0.96	*trnS-GGA*	Trp	UGG	448	**1.00**	*trnW-CCA*
Ser	UCA	394	**1.20**	*trnS-UGA*	Arg	CGU	336	**1.32**	*trnR-ACG*
Ser	UCG	154	0.47		Arg	CGC	100	0.39	
Ser	AGA	474	**1.86**		Arg	CGA	339	**1.33**	
Ser	AGG	165	0.65	*trnS-GCU*	Arg	CGG	118	0.46	
Tyr	UAU	799	**1.64**		Arg	AGU	406	**1.24**	*trnR-UCU*
Tyr	UAC	175	0.36	*trnY-GUA*	Arg	AGC	118	0.36	
Pro	CCA	322	**1.17**	*trnP-UGG*	Gly	GGU	581	**1.32**	
Pro	CCG	159	0.58		Gly	GGC	188	0.43	*trnG-GCC*
Pro	CCU	434	**1.58**		Gly	GGA	686	**1.56**	*trnG-UCC*
Pro	CCC	184	0.67		Gly	GGG	305	0.69	
Thr	ACU	529	**1.64**		Ala	GCA	416	**1.18**	*trnA-UGC*
Thr	ACC	236	0.73	*trnT-GGU*	Ala	GCG	162	0.46	
Thr	ACA	404	**1.25**	*trnT-UGU*	Ala	GCU	611	**1.73**	
Thr	ACG	125	0.39		Ala	GCC	223	0.63	

RSCU: Relative synonymous codon usage. RSCU > 1 are highlighted in bold.

**Table 5:** The codon-anticodon recognition pattern and codon usage for the *K. indica* cp genome.

Amino Acid	Codon	No.	RSCU	tRNA	Amino Acid	Codon	No.	RSCU	tRNA
Phe	UUU	982	**1.31**		Stop	UAA	49	**1.73**	
Phe	UUC	515	0.69	*trnF-GAA*	Stop	UAG	21	0.74	
Leu	UUA	870	**1.87**	*trnL-UAA*	Stop	UGA	15	0.53	
Leu	UUG	578	**1.24**	*trnL-CAA*	His	CAU	452	**1.51**	
Leu	CUU	607	**1.30**		His	CAC	148	0.49	*trnH-GUG*
Leu	CUC	186	0.4		Gln	CAA	723	**1.53**	*trnQ-UUG*
Leu	CUA	375	0.81	*trnL-UAG*	Gln	CAG	220	0.47	
Leu	CUG	179	0.38		Asn	AAU	969	**1.53**	
Ile	AUU	1072	**1.47**		Asn	AAC	295	0.47	*trnN-GUU*
Ile	AUC	427	0.58	*trnI-GAU*	Lys	AAA	1036	**1.48**	*trnK-UUU*
Ile	AUA	691	0.95	*trnI-CAU*	Lys	AAG	360	0.52	
Met	AUG	633	1	*trn(f)M-CAU*	Asp	GAU	847	**1.61**	
Val	GUU	503	**1.45**		Asp	GAC	205	0.39	*trnD-GUC*
Val	GUC	180	0.52	*trnV-GAC*	Glu	GAA	987	**1.47**	*trnE-UUC*
Val	GUA	517	**1.49**	*trnV-UAC*	Glu	GAG	359	0.53	
Val	GUG	190	0.55		Cys	UGU	209	**1.42**	
Ser	UCU	585	**1.76**		Cys	UGC	85	0.58	*trnC-GCA*
Ser	UCC	308	0.93	*trnS-GGA*	Trp	UGG	463	1	*trnW-CCA*
Ser	UCA	401	**1.21**	*trnS-UGA*	Arg	CGU	351	**1.33**	*trnR-ACG*
Ser	AGA	488	**1.85**		Arg	CGC	108	0.41	
Ser	AGG	177	0.67	*trnS-GCU*	Arg	CGA	346	**1.31**	
Ser	UCG	172	0.52		Arg	CGG	114	0.43	
Tyr	UAU	804	**1.64**		Arg	AGU	405	**1.22**	*trnR-UCU*
Tyr	UAC	175	0.36	*trnY-GUA*	Arg	AGC	121	0.36	
Pro	CCU	414	**1.50**		Gly	GGU	571	**1.28**	
Pro	CCC	209	0.76		Gly	GGC	199	0.45	*trnG-GCC*
Pro	CCA	314	**1.14**	*trnP-UGG*	Gly	GGA	681	**1.53**	*trnG-UCC*
Pro	CCG	167	0.61		Gly	GGG	328	0.74	
Thr	ACU	531	**1.62**		Ala	GCU	622	**1.74**	
Thr	ACC	239	0.73	*trnT-GGU*	Ala	GCC	233	0.65	
Thr	ACA	405	**1.23**	*trnT-UGU*	Ala	GCA	410	**1.15**	*trnA-UGC*
Thr	ACG	137	0.42		Ala	GCG	161	0.45	

**Table 6:** The repeats of the *C. carinatum* Schousb cp genome and their distribution.

No.	Size (bp)	Type	Repeat 1 Location	Repeat 2 Location	Region	No.	Size (bp)	Type	Repeat 1 Location	Repeat 2 Location	Region
1	60	F	*ycf2* (CDS)	*ycf2* (CDS)	IRb	22	31	R	IGS (*trnT-GGU, psbD*)	IGS (*trnT-GGU, psbD*)	LSC
2	60	P	*ycf2* (CDS)	*ycf2* (CDS)	IRb, IRa	23	30	P	IGS (*psbI, trnS-GUC*)	*trnS-GGA* (CDS)	LSC
3	60	P	*ycf2* (CDS)	*ycf2* (CDS)	IRb, IRa	24	30	F	*ycf2* (CDS)	*ycf2* (CDS)	IRb
4	60	F	*ycf2* (CDS)	*ycf2* (CDS)	IRa, IRa	25	30	P	*ycf2* (CDS)	*ycf2* (CDS)	IRb, IRa
5	51	F	IGS (*rps11, rpl36*)	IGS (*rps11, rpl36*)	LSC	26	30	P	*ycf2* (CDS)	*ycf2* (CDS)	IRb, IRa
6	48	P	IGS (*psbT, psbN*)	IGS (*psbT, psbN*)	LSC	27	35	F	*psaB* (CDS)	*psaA* (CDS)	LSC
7	46	F	IGS (*accD, psaI*)	IGS (*accD, psaI*)	LSC	28	35	F	*ycf3* (intron2)	*ndhB* (intron)	LSC, IRb
8	45	F	*ycf2* (CDS)	*ycf2* (CDS)	IRb	29	35	P	*ycf3* (intron2)	*ndhB* (intron)	LSC, IRa
9	45	P	*ycf2* (CDS)	*ycf2* (CDS)	IRb, IRa	30	32	P	IGS (*trnT-GGU, psbD*)	IGS (*trnT-GGU, psbD*)	LSC
10	45	P	*ycf2* (CDS)	*ycf2* (CDS)	IRb, IRa	31	31	F	IGS (*psbI, trnS-GUC*)	IGS (*psbI, trnS-GUC*)	LSC
11	46	P	IGS (*petN, psbM*)	IGS (*petN, psbM*)	LSC	32	30	P	IGS (*psbC, trnS-UGA*)	*trnS-GGA* (CDS)	LSC
12	39	P	IGS (*rps12, trnV-GAC*)	*ndhA* (intron)	IRb, SSC	33	30	F	*rbcL* (CDS)	IGS (*rbcL, accD*)	LSC
13	39	F	*ndhA* (intron)	IGS (*trnV-GAC, rps12*)	SSC, IRa	34	32	F	IGS (*psbI, trnS-GUC*)	IGS (*psbC, trnS-UGA*)	LSC
14	41	F	*ycf3* (intron2)	IGS (*rps12, trnV-GAC*)	LSC, IRb	35	31	R	IGS (*trnL-UAG, rpl32*)	IGS (*trnL-UAG, rpl32*)	SSC
15	41	P	*ycf3* (intron2)	IGS (*trnV-GAC, rps12*)	LSC, IRa	36	30	C	IGS (*trnT-GGU, psbD*)	IGS (*trnT-GGU, psbD*)	LSC
16	39	P	*ycf3* (intron2)	*ndhA* (intron)	LSC, SSC	37	30	F	*psaB* (CDS)	*psaA* (CDS)	LSC
17	42	F	*ycf2* (CDS)	*ycf2* (CDS)	IRb	38	30	F	*ycf2* (CDS)	*ycf2* (CDS)	IRb, IRa
18	42	P	*ycf2* (CDS)	*ycf2* (CDS)	IRb, IRa	39	30	P	*ycf2* (CDS)	*ycf2* (CDS)	IRb
19	42	P	*ycf2* (CDS)	*ycf2* (CDS)	IRb, IRa	40	30	F	IGS (*trnL-UAG, rpl32*)	IGS (*trnL-UAG, rpl32*)	SSC
20	42	F	*ycf2* (CDS)	*ycf2* (CDS)	IRa	41	30	R	IGS (*trnL-UAG, rpl32*)	IGS (*trnL-UAG, rpl32*)	SSC
21	41	P	IGS (*ndhE, psaC*)	IGS (*psaC, ndhD*)	SSC	42	30	P	*ycf2* (CDS)	*ycf2* (CDS)	IRa

F = forward, P = palindrome, R = reverse, C = complement, IGS = intergenic spacer.

**Table 7:** The repeats of the *K. indica* cp genome and their distribution.

No.	Size (bp)	Type	Repeat 1 Location	Repeat 2 Location	Region	No.	Size (bp)	Type	Repeat 1 Location	Repeat 2 Location	Region
1	48	P	IGS (*psbT, psbN*)	IGS (*psbT, psbN*)	LSC	31	32	F	IGS (*rrn5, rrn4.5*)	IGS (*rrn5, rrn4.5*)	IRa
2	39	P	*ycf3* (intron2)	*ndhA* (intron)	LSC, SSC	32	34	R	IGS (*trnT-GGU, psbD*)	IGS (*trnT-GGU, psbD*)	LSC
3	41	F	*ycf3* (intron2)	IGS (*rps12, trnV-GAC*)	LSC, IRb	33	31	R	IGS (*ndhC, trnV-UAC*)	*petB* (intron)	LSC
4	41	P	*ycf3* (intron2)	IGS (*trnV-GAC, rps12*)	LSC, IRa	34	33	C	IGS (*accD, psaI*)	*petD* (CDS)	LSC
5	39	P	IGS (*rps12, trnV-GAC*)	*ndhA* (intron)	IRb, SSC	35	33	C	IGS (*accD, psaI*)	IGS (*accD, psaI*)	LSC
6	39	F	*ndhA* (intron)	IGS (*trnV-GAC, rps12*)	SSC, IRa	36	30	P	IGS (*psbC, trnS-UGA*)	*trnS-GGA* (CDS)	LSC
7	42	F	*ycf2* (CDS)	*ycf2* (CDS)	IRb	37	32	C	IGS (*trnH-GUG, psbA*)	IGS (*accD, psaI*)	LSC
8	42	P	*ycf2* (CDS)	*ycf2* (CDS)	IRb, IRa	38	32	F	IGS (*psbI, trnS-GCU*)	IGS (*psbC, trnS-UGA*)	LSC
9	42	P	*ycf2* (CDS)	*ycf2* (CDS)	IRb, IRa	39	32	P	IGS (*petN, psbM*)	IGS (*petN, psbM*)	LSC
10	42	F	*ycf2* (CDS)	*ycf2* (CDS)	IRa	40	32	C	IGS (*trnG-UCC, trnT-GGU*)	*petD* (intron)	LSC
11	41	R	*petD* (intron)	*petD* (intron)	LSC	41	32	F	*psaB* (CDS)	*psaA* (CDS)	LSC
12	43	P	IGS (*psaC, ndhD*)	IGS (*psaC, ndhD*)	SSC	42	32	R	IGS (*accD, psaI*)	IGS (*accD, psaI*)	LSC
13	39	R	IGS (*accD, psaI*)	IGS (*accD, psaI*)	LSC	43	31	P	IGS (*trnG-UCC, trnT-GGU*)	IGS (*ndhC, trnV-UAC*)	LSC
14	31	F	IGS (*rps12, trnV-GAC*)	IGS (*rps12, trnV-GAC*)	IRb	44	31	P	IGS (*trnG-UCC, trnT-GGU*)	IGS (*trnG-UCC, trnT-GGU*)	LSC
15	31	P	IGS (*rps12, trnV-GAC*)	IGS (*trnV-GAC, rps12*)	IRb, IRa	45	31	P	IGS (*trnT-GGU, psbD*)	IGS (*trnT-UGU, trnL-UAA*)	LSC
16	31	P	IGS (*rps12, trnV-GAC*)	IGS (*trnV-GAC, rps12*)	IRb, IRa	46	31	F	IGS (*psaA, ycf3*)	IGS (*psaA, ycf3*)	LSC
17	31	F	IGS (*trnV-GAC, rps12*)	IGS (*trnV-GAC, rps12*)	IRa	47	31	R	IGS (*accD, psaI*)	IGS (*accD, psaI*)	LSC
18	37	P	IGS (*rpl22, rps19*)	IGS (*rpl22, rps19*)	LSC	48	31	F	IGS (*accD, psaI*)	*petD* (intron)	LSC
19	31	R	*petD* (intron)	*petD* (intron)	LSC	49	31	C	*petD* (intron)	*petD* (intron)	LSC
20	30	P	IGS (*psbI, trnS-GCU*)	*trnS-GGA* (CDS)	LSC	50	31	F	*ndhF* (CDS)	IGS (*ndhF, ycf1*)	SSC
21	30	F	*ycf2* (CDS)	*ycf2* (CDS)	IRb	51	30	C	IGS (*trnG-UCC, trnT-GGU*)	IGS (*trnT-GGU, psbD*)	LSC
22	30	P	*ycf2* (CDS)	*ycf2* (CDS)	IRb, IRa	52	30	R	IGS (*trnT-GGU, psbD*)	IGS (*trnT-GGU, psbD*)	LSC
23	30	P	*ycf2* (CDS)	*ycf2* (CDS)	IRb, IRa	53	30	C	*ycf3* (intron2)	IGS (*ndhC, trnV-UAC*)	LSC
24	32	P	IGS (*trnG-UCC, trnT-GGU*)	IGS (*trnG-UCC, trnT-GGU*)	LSC	54	30	C	IGS (*ndhC, trnV-UAC*)	IGS (*accD, psaI*)	LSC
25	32	P	IGS (*psbZ, trnG-GCC*)	IGS (*psbZ, trnG-GCC*)	LSC	55	30	F	IGS (*ndhC, trnV-UAC*)	IGS (*ndhC, trnV-UAC*)	LSC
26	32	P	IGS (*accD, psaI*)	*petD* (CDS)	LSC	56	30	F	IGS (*accD, psaI*)	IGS (*accD, psaI*)	LSC
27	32	R	*petD* (CDS)	*petD* (CDS)	LSC	57	30	R	IGS (*accD, psaI*)	IGS (*accD, psaI*)	LSC
28	32	F	IGS (*rrn4.5, rrn5*)	IGS (*rrn4.5, rrn5*)	IRb	58	30	R	IGS (*accD, psaI*)	*petD* (intron)	LSC
29	32	P	IGS (*rrn4.5, rrn5*)	IGS (*rrn5, rrn4.5*)	IRb, IRa	59	30	F	*petD* (intron)	*petD* (intron)	LSC
30	32	P	IGS (*rrn4.5, rrn5*)	IGS (*rrn5, rrn4.5*)	IRb, IRa						

F = forward, P = palindrome, R = reverse, C = complement, IGS = intergenic spacer.

**Table 8:** The simple sequence repeats in the *C. carinatum* Schousb cp genome.

Unit	Length	No.	Location	Region	Unit	Length	No.	Location	Region	Unit	Length	No.	Location	Region
A	19	1	IGS (*rpl32, ndhF*)	SSC	T	22	1	IGS (*atpF, atpA*)	LSC	TA	14	1	IGS (*trnH-GUG, psbA*)	LSC
	13	1	IGS (*psbE, petL*)	LSC		16	1	*rps16* (intron)	LSC		12	2	IGS (*trnT-GGU, psbD*)	LSC
	12	1	IGS (*ycf1, rps15*)	SSC		14	3	IGS (*trnE-UUC, rpoB*)	LSC				IGS (*rpl33, rps18*)	LSC
	11	6	IGS (*trnK-UUU, rps16*)	LSC				IGS (*atpB, rbcL*)	LSC		10	1	*rpoC1* (exonII)	LSC
			IGS (*trnR-UCU, trnG-UCC*)	LSC				IGS (*rps8, rpl14*)	LSC	TG	10	1	IGS (*rps16, trnQ-UUG*)	LSC
			IGS (*psbZ, trnG-GCC*)	LSC		13	3	IGS (*ndhC, trnV-UAC*)	LSC	ATT	12	2	IGS (*cemA, petA*)	LSC
			IGS (*psbE, petL*)	LSC				IGS (*rbcL, accD*)	LSC				IGS (*trnL-UAG, rpl32*)	SSC
			IGS (*rps18, rpl20*)	LSC				IGS (*psbB, psbT*)	LSC	GAA	15	1	*ycf1*	SSC
			IGS (*petD, rpoA*)	LSC		12	5	IGS (*trnH-GUG, psbA*)	LSC	TTA	12	1	*ndhA* (intron)	SSC
	10	10	*trnk-UUU* (intron)	LSC				IGS (*trnD-GUC, trnY-GUA*)	LSC	TTC	12	1	*psbC*	LSC
			*rpoB*	LSC				*ycf3* (intronII)	LSC	AATA	12	2	IGS (*trnK-UUU, rps16*)	LSC
			*rpoC1* (intron)	LSC				*clpP* (intronI)	LSC				*ndhA* (intron)	SSC
			*rpoC1* (exonII)	LSC				*ycf1*	LSC	ATAC	12	1	IGS (*trnF-GAA, ndhJ*)	LSC
			*rpoC1* (exonII)	LSC		11	1	IGS (*petA, psbJ*)	LSC	ATTG	12	1	*ycf2*	IRa
			IGS (*psaA, ycf3*)	LSC		10	9	*trnk-UUU* (intron)	LSC	ATTT	22	1	IGS (*atpF, atpA*)	LSC
			IGS (*ycf3, trnS-GGA*)	LSC				IGS (*psbM, trnD-GUC*)	LSC	CAAT	12	1	IGS (*ycf2, trnL-CAA*)	IRb
			IGS (*trnT-UGU, rnL-UAA*)	LSC				*rpoC1* (intron)	LSC	GATT	12	1	*ndhA* (intron)	SSC
			*psbT*	LSC				IGS (*atpI, atpH*)	LSC	TAGA	12	1	IGS (*rbcL, accD*)	LSC
			IGS (*rrn5, trnR-ACG*)	IRb				IGS (*atpI, atpH*)	LSC	TAAA	12	1	IGS (*atpI, atpH*)	LSC
C	12	1	*rps16* (intron)	LSC				IGS (*atpA, trnR-UCU*)	LSC	TAAT	12	1	IGS (*petN, psbM*)	LSC
AT	14	1	*rps16* (intron)	LSC				*rpoA*	LSC	TATT	12	2	IGS (*rpl33, rps18*)	LSC
	12	1	IGS (*psbZ, trnG-GCC*)	LSC				IGS (*rpl14, rpl16*)	LSC				*ndhD*	SSC
	10	1	*rpoC2*	LSC				IGS (*trnR-ACG, rrn5*)	IRa	TTTC	16	1	*rpl16* (intron)	LSC
										AATTT	15	1	IGS (*ccsA, trnL-UAG*)	SSC

**Table 9:** The simple sequence repeats in the *K. indica* cp genome.

Unit	Length	No.	Location	Region	Unit	Length	No.	Location	Region	Unit	Length	No.	Location	Region
A	18	1	IGS (*trnS-UGA, psbZ*)	LSC	AT	16	1	IGS (*psbZ, trnG-GCC*)	LSC	GAA	15	1	*ycf1*	SSC
	12	1	IGS (*psbZ, trnG-GCC*)	LSC		10	7	*rps16* (intron)	LSC		12	1	*ycf1*	SSC
	11	3	IGS (*trnQ-UUG, psbK*)	LSC		10		*rpoC2*	LSC	TAT	21	1	IGS (*rps12, trnV-GAC*)	IRb
	11		IGS (*psbM, trnD-GUC*)	LSC		10		IGS (*psbZ, trnG-GCC*)	LSC		12	2	IGS (*trnT-UGU, trnL-UAA*)	LSC
	11		IGS (*ycf4, cemA*)	LSC		10		IGS (*psbZ, trnG-GCC*)	LSC		12		IGS (*trnF-GAA, ndhJ*)	LSC
	10	7	*rpoB*	LSC		10		IGS (*psbE, petL*)	LSC	TTA	15	2	IGS (*ndhC, trnV-UAC*)	LSC
	10		*rpoC1* (exonII)	LSC		10		IGS (*petD, rpoA*)	LSC		15		IGS (*clpP, psbB*)	LSC
	10		IGS (*petB, petD*)	LSC		10		*rpl16* (intron)	LSC		12	2	*petB* (intron)	LSC
	10		*ndhB* (intron)	IRb	TA	23	1	*petD* (intron)	LSC		12		*petD* (intron)	LSC
	10		*ndhA* (intron)	SSC		20	1	IGS (*accD, psaI*)	LSC		12	1	*psbC*	LSC
	10		IGS (*trnL-UAG, rpl32*)	SSC		18	1	*petD* (intron)	LSC		12	1	IGS (*trnK-UUU, rps16*)	LSC
	10		IGS (*rpl2, rps19*)	IRa		14	1	IGS (*accD, psaI*)	LSC		12	1	IGS (*trnG-UCC, trnT-GGU*)	LSC
T	18	1	IGS (*trnE-UUC, rpoB*)	LSC		12	3	IGS (*trnT-GGU, psbD*)	LSC		12	1	IGS (*trnE-UUC, rpoB*)	LSC
	17	1	*rpoA*	LSC		12		*petD* (intron)	LSC		12	1	IGS (*clpP, psbB*)	LSC
	14	2	IGS (*rpl20, rps12*)	LSC		12		IGS (*rpl22, rps19*)	LSC		12	1	IGS (*trnS-GCU, trnC-GCA*)	LSC
	14		*ndhA* (intron)	SSC		10	3	*rpoC1* (exonII)	LSC		12	1	IGS (*ndhI, ndhG*)	SSC
	13	1	IGS (*psbE, petL*)	LSC		10		IGS (*psaJ, rpl33*)	LSC	TATC	12	1	IGS (*psbA, trnK-UUU*)	LSC
	12	2	IGS (*atpF, atpA*)	LSC		10		*ndhA* (intron)	SSC	TATT	12	2	IGS (*rpl33, rps18*)	LSC
	12		*clpP* (intronII)	LSC	AAT	12	2	IGS (*trnR-UCU, trnG-UCC*)	LSC		12		IGS (*psaC, ndhD*)	SSC
	11	2	IGS (*atpB, rbcL*)	LSC		12		IGS (*trnT-GGU, psbD*)	LSC	TCTA	12	1	*ndhA* (intron)	SSC
	11		IGS (*psaI, ycf4*)	LSC	ATA	21	1	IGS (*trnV-GAC, rps12*)	IRa	TTCT	12	1	IGS (*trnG-UCC, trnT-GGU*)	LSC
	10	9	IGS (*trnC-GCA, petN*)	LSC		15	3	*ycf3* (intron)	LSC	TTTA	12	1	*petD* (intron)	LSC
	10		IGS (*rpoC2, rps2*)	LSC		15		IGS (*trnP-UGG, psaJ*)	LSC	TTTC	12	1	*trnK-UUU* (intron)	LSC
	10		IGS (*atpI, atpH*)	LSC		15		IGS (*trnL-UAG, rpl32*)	SSC	ATTAG	15	1	IGS (*rbcL, accD*)	LSC
	10		IGS (*ndhC, trnV-UAC*)	LSC		12	2	IGS (*trnG-UCC, trnT-GGU*)	LSC	TATAT	23	1	*petD* (intron)	LSC
	10		*clpP* (intronI)	LSC		12		*rpl16* (intron)	LSC		20	1	IGS (*accD, psaI*)	LSC
	10		IGS (*rps8, rpl14*)	LSC	ATT	15	1	IGS (*trnG-UCC, trnT-GGU*)	LSC		15	1	IGS (*accD, psaI*)	LSC
	10		IGS (*rps19, rpl2*)	IRb	TAA	15	1	IGS (*accD, psaI*)	LSC	TATTA	21	2	IGS (*rps12, trnV-GAC*)	IRb
	10		IGS (*psaC, ndhD*)	SSC		12	2	IGS (*trnE-UUC, rpoB*)	LSC		21		IGS (*trnV-GAC, rps12*)	IRa
	10		*ndhB* (intron)	IRa		12		IGS (*trnT-GGU, psbD*)	LSC	TCCTA	15	1	IGS (*rps4, trnT-UGU*)	LSC

**Table 10:** The distribution of SSRs present in the *Asteraceae* cp genomes.

Taxon	Genome Size (bp)	GC (%)	SSR Type							CDS		
			Mono	Di	Tri	Tetra	Penta	Hexa	Total	% ^a^	No. ^b^	% ^c^
*C* *. carinatum Schousb*	149,752	37.47	43	8	5	13	1	0	70	51.6	12	17.1
*K* *. indica*	152,885	37.25	30	18	22	13	7	0	90	51.3	9	10
*D* *. alveolatum*	152,265	37.37	34	14	11	14	2	0	75	51.4	8	10.7
*A* *. annua*	150,952	37.48	39	10	4	15	3	0	71	52.1	10	14.1
*E* *. paradoxa*	151,837	37.59	37	4	4	5	0	1	51	51.5	14	27.5
*H* *. annuus*	151,104	37.62	40	4	4	4	0	0	52	51.2	14	26.9
*T* *. mongolicum*	151,451	37.67	14	5	3	6	1	1	30	51.3	11	36.7
*C* *. indicum*	150,972	37.48	38	10	4	14	1	0	67	48.8	7	10.4

CDS: protein-coding regions. ^a^ the percentage ratio of the total length of the CDS to the genome size. ^b^ the total number of SSRs in CDS. ^c^ the percentage ratio of the total number of SSRs in CDS to the total number of SSRs in the whole genome.

## Supplementary Materials

Supplementary materials are available online.
