# Supplementary material for: Sequencing and Analysis of Chrysanthemum carinatum Schousb and Kalimeris indica. The Complete Chloroplast Genomes Reveal Two Inversions and rbcL as Barcoding of the Vegetable
Source: Molecules. 2018 Jun 5;23(6):1358. doi: 10.3390/molecules23061358 (PMC6099409; doi:10.3390/molecules23061358)
Supplement: Supplementary file 1 [file molecules-23-01358-s001.pdf]

Sequencing and Analysis of *Chrysanthemum carinatum* Schousb and *Kalimeris indica*. The Complete Chloroplast Genomes Reveal Two Inversions and *rbcL* as Barcoding of the Vegetable

Supplemental Materials:

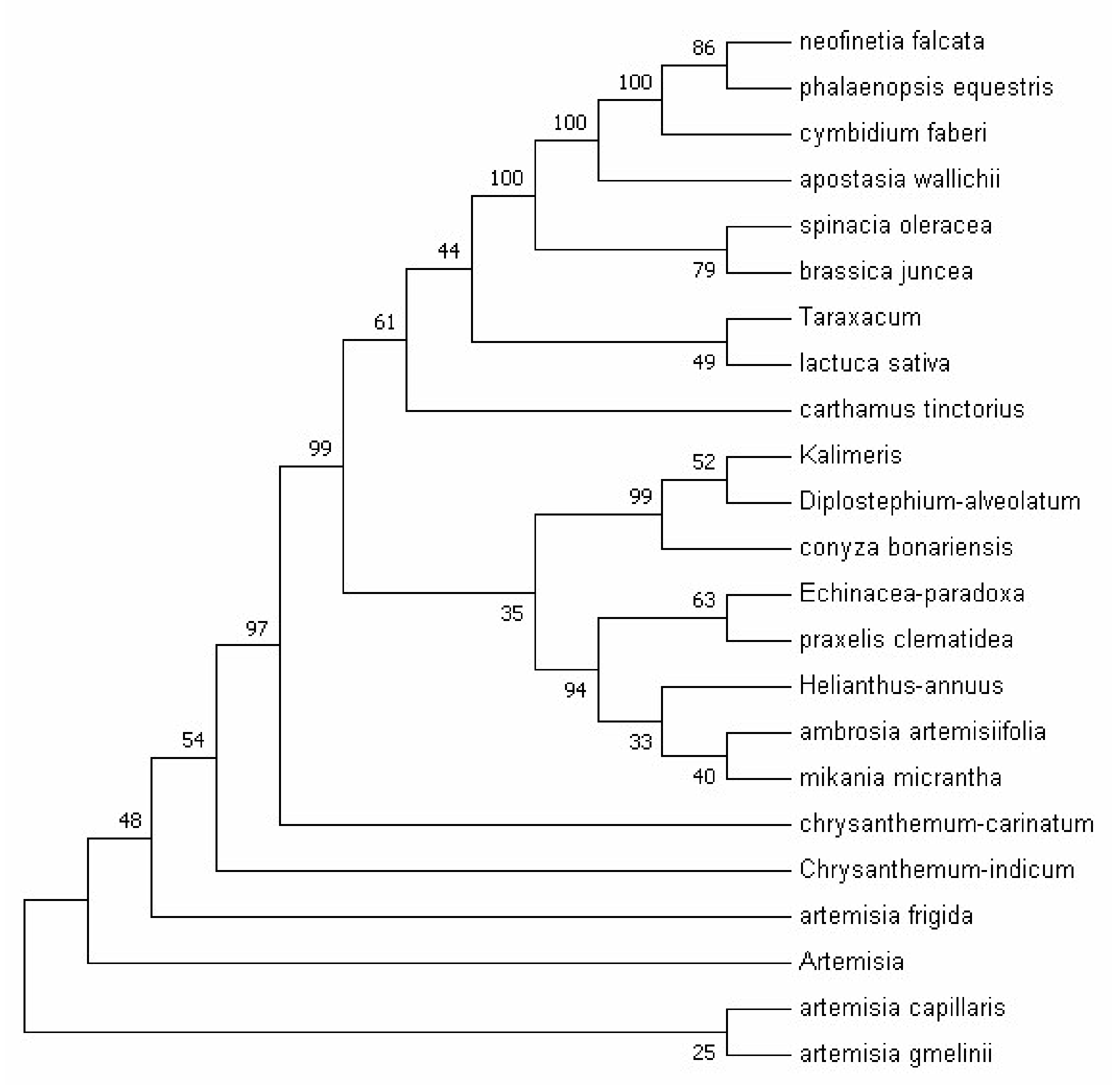

Figure S1. The molecular phylogenetic analysis of the cp protein-coding gene *atpF* for 24 samples using the Maximum Likelihood method. The tree was constructed by using MEGA7 . The stability of each tree node was tested by bootstrap analysis with 1000 replicates.

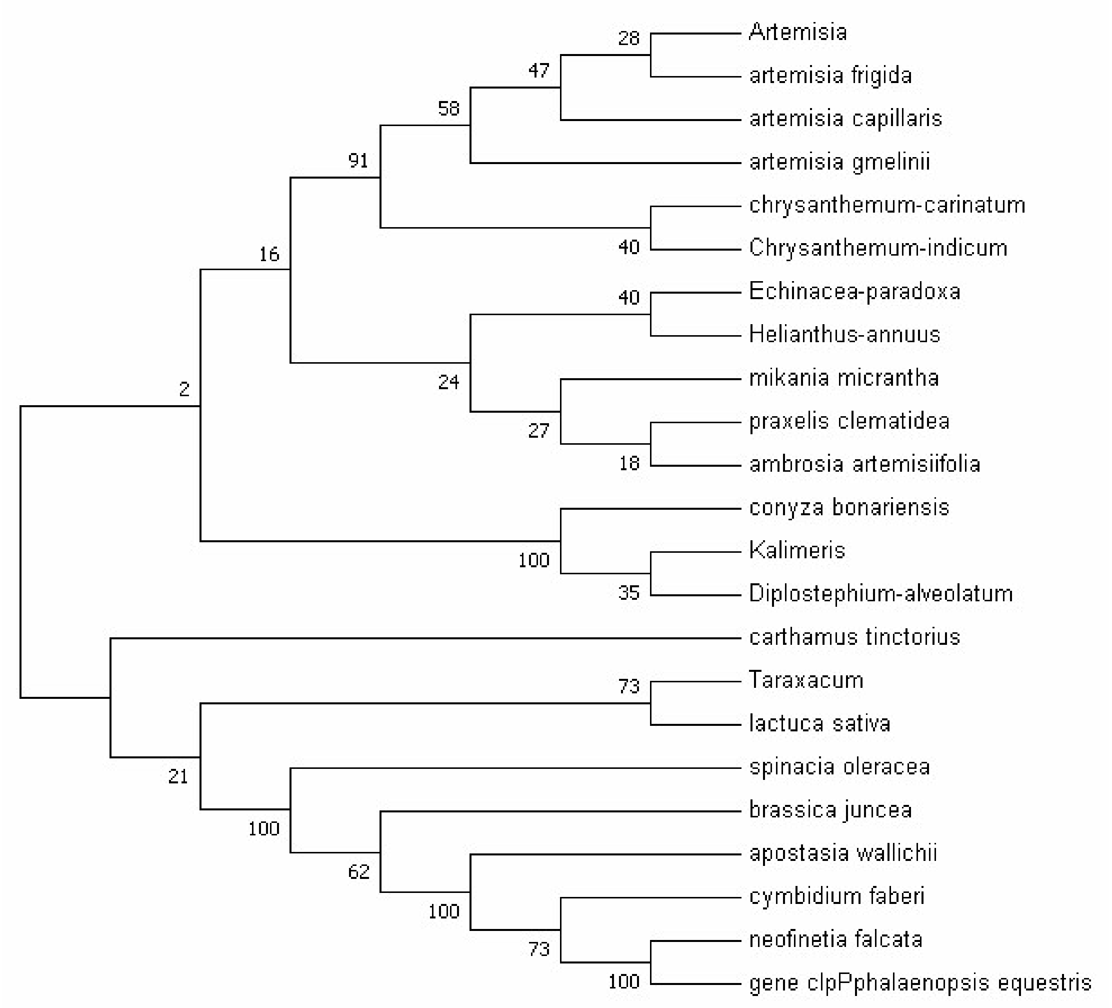

Figure S2. The molecular phylogenetic analysis of the cp protein-coding gene *clpP* for 24 samples using the Maximum Likelihood method. The tree was constructed by using MEGA7 . The stability of each tree node was tested by bootstrap analysis with 1000 replicates.

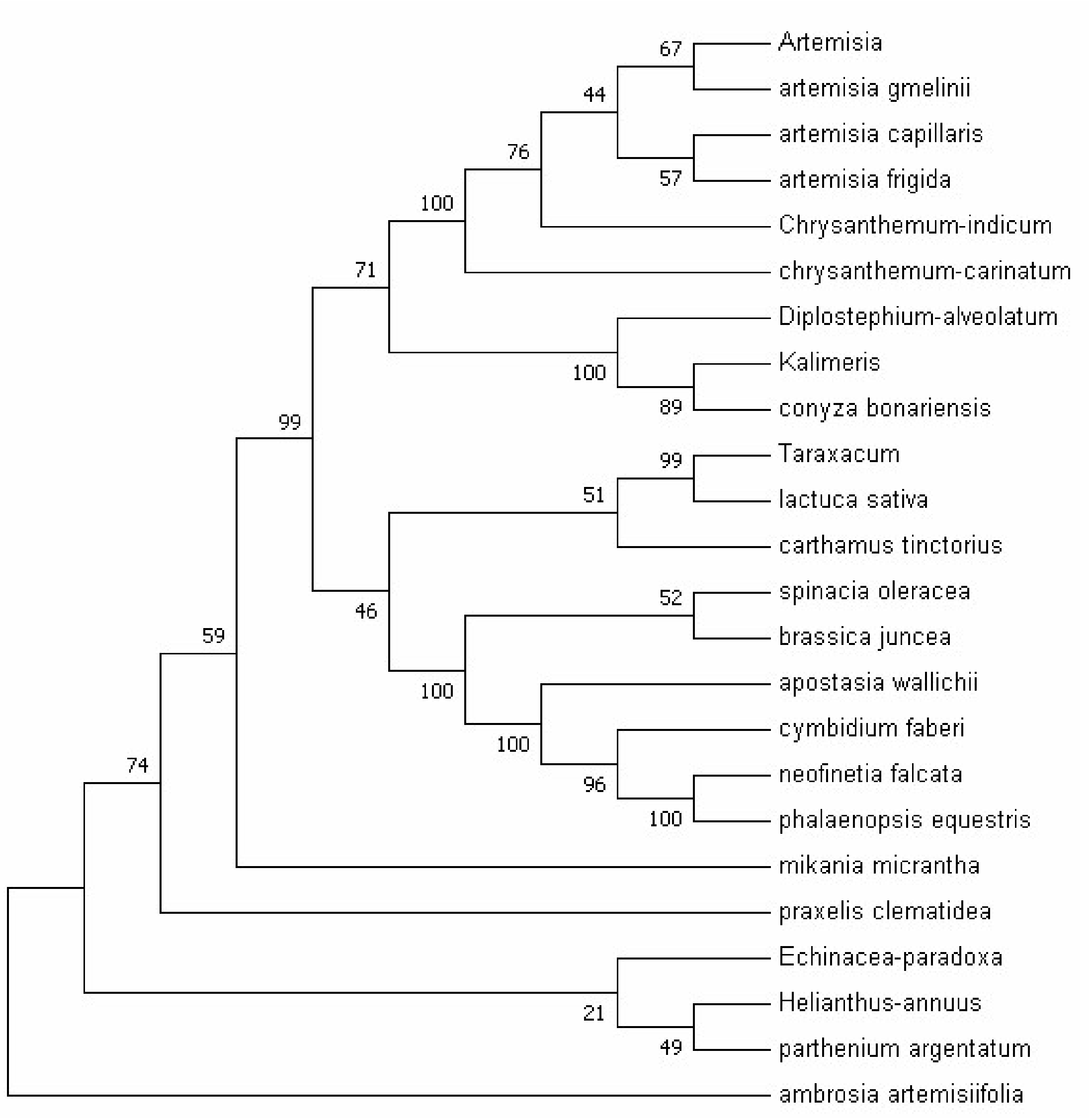

Figure S3. The molecular phylogenetic analysis of the cp protein-coding gene *matK* for 24 samples using the Maximum Likelihood method. The tree was constructed by using MEGA7 . The stability of each tree node was tested by bootstrap analysis with 1000 replicates.

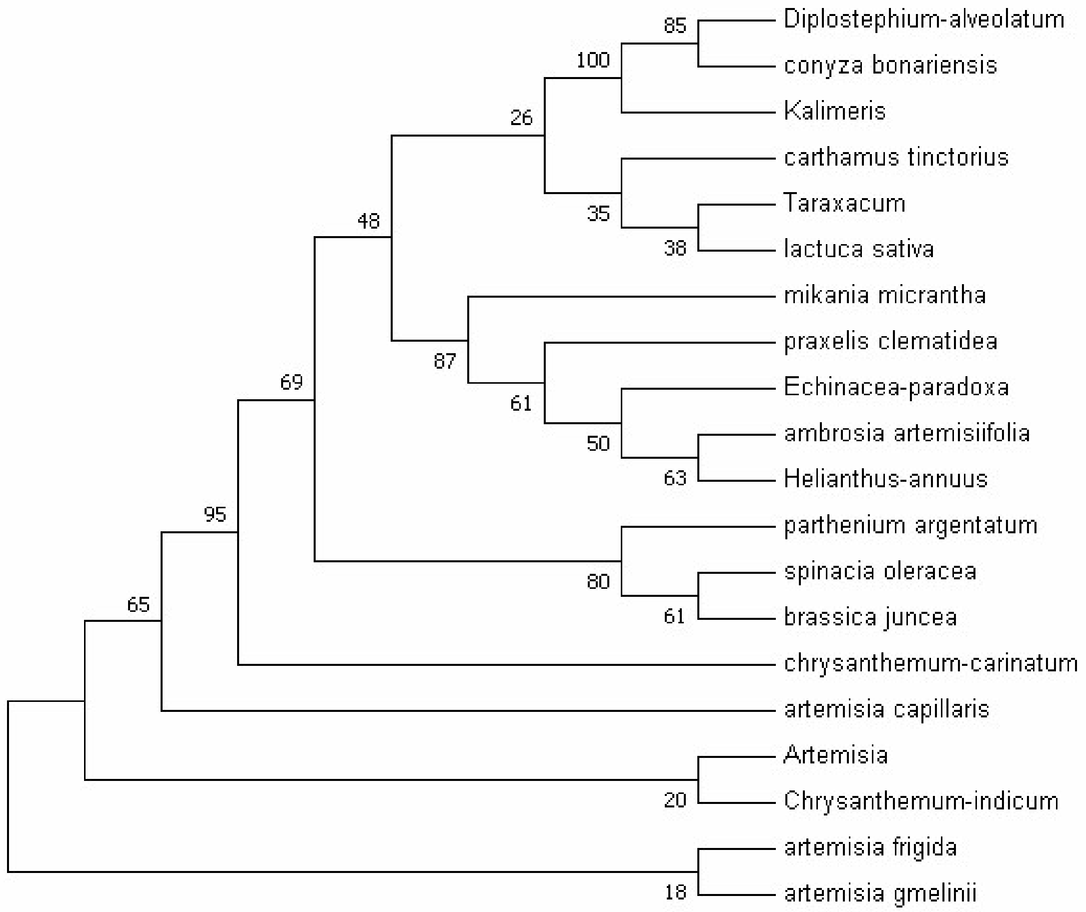

Figure S4. The molecular phylogenetic analysis of the cp protein-coding gene *ndhA* for 24 samples using the Maximum Likelihood method. The tree was constructed by using MEGA7 . The stability of each tree node was tested by bootstrap analysis with 1000 replicates.

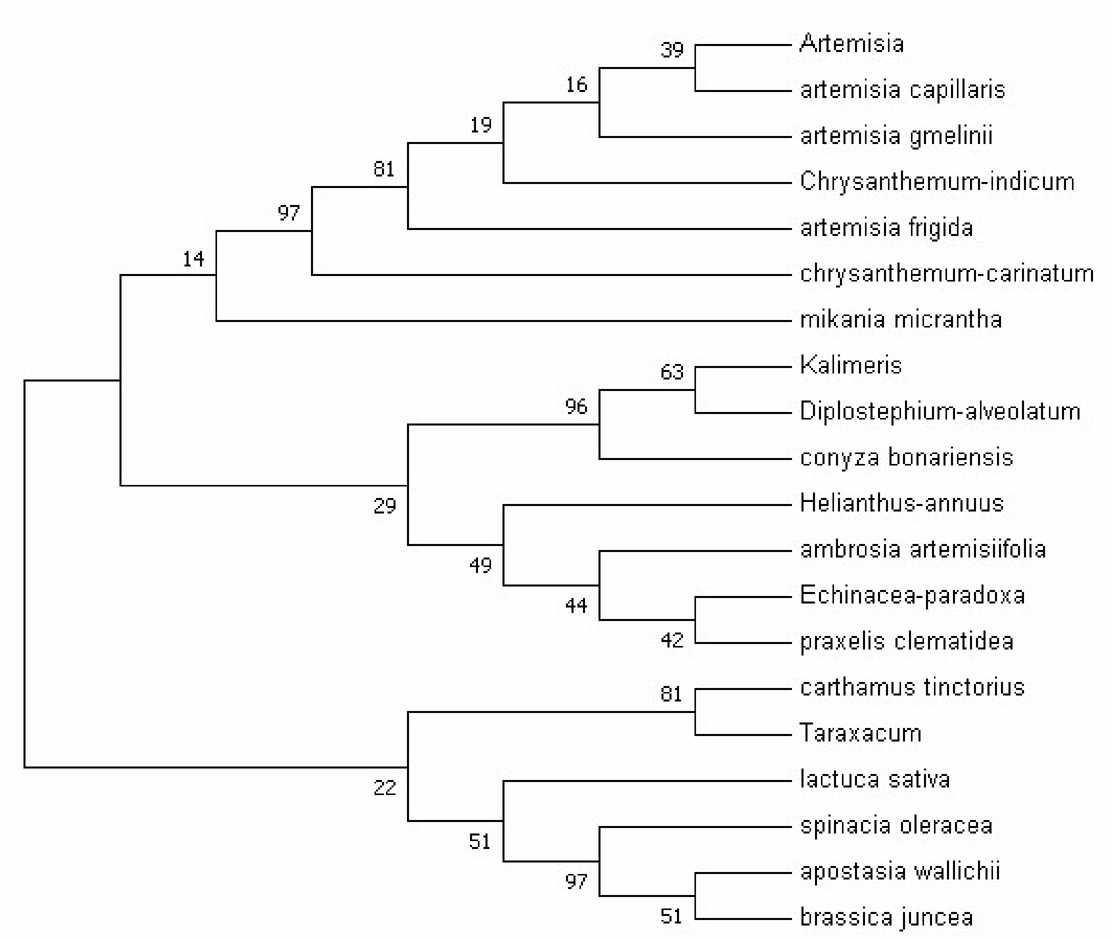

Figure S5. The molecular phylogenetic analysis of the cp protein-coding gene *ndhB* for 24 samples using the Maximum Likelihood method. The tree was constructed by using MEGA7 . The stability of each tree node was tested by bootstrap analysis with 1000 replicates.

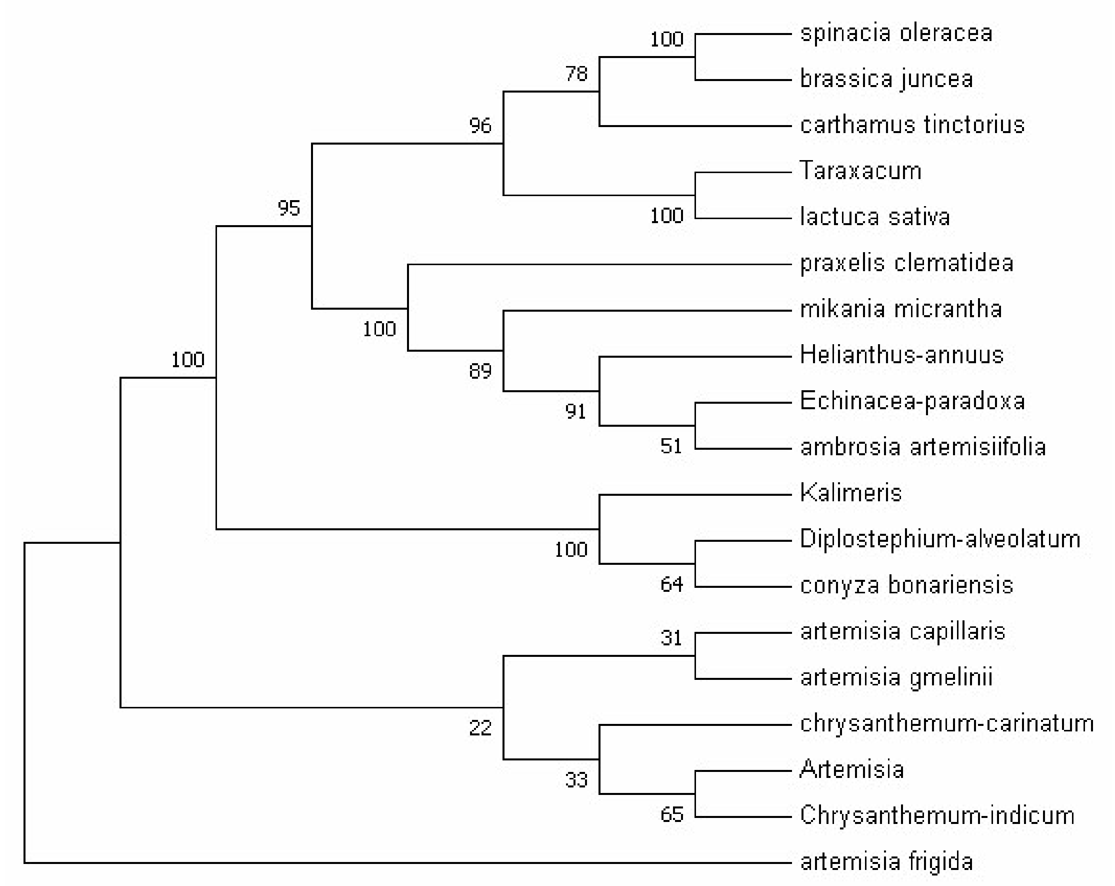

Figure S6. The molecular phylogenetic analysis of the cp protein-coding gene *ndhF* for 24 samples using the Maximum Likelihood method. The tree was constructed by using MEGA7 . The stability of each tree node was tested by bootstrap analysis with 1000 replicates.

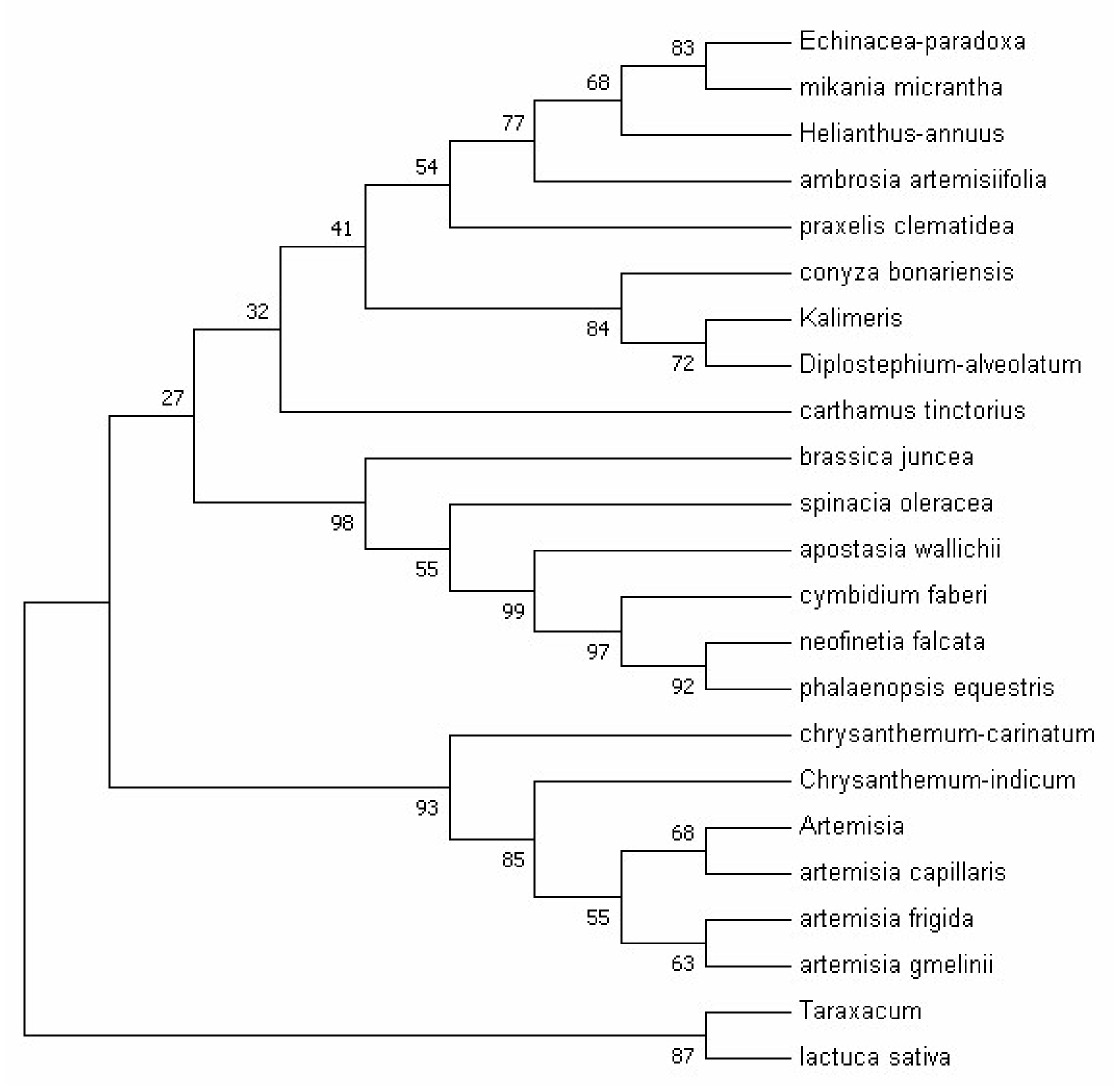

Figure S7. The molecular phylogenetic analysis of the cp protein-coding gene *petB* for 24 samples using the Maximum Likelihood method. The tree was constructed by using MEGA7 . The stability of each tree node was tested by bootstrap analysis with 1000 replicates.

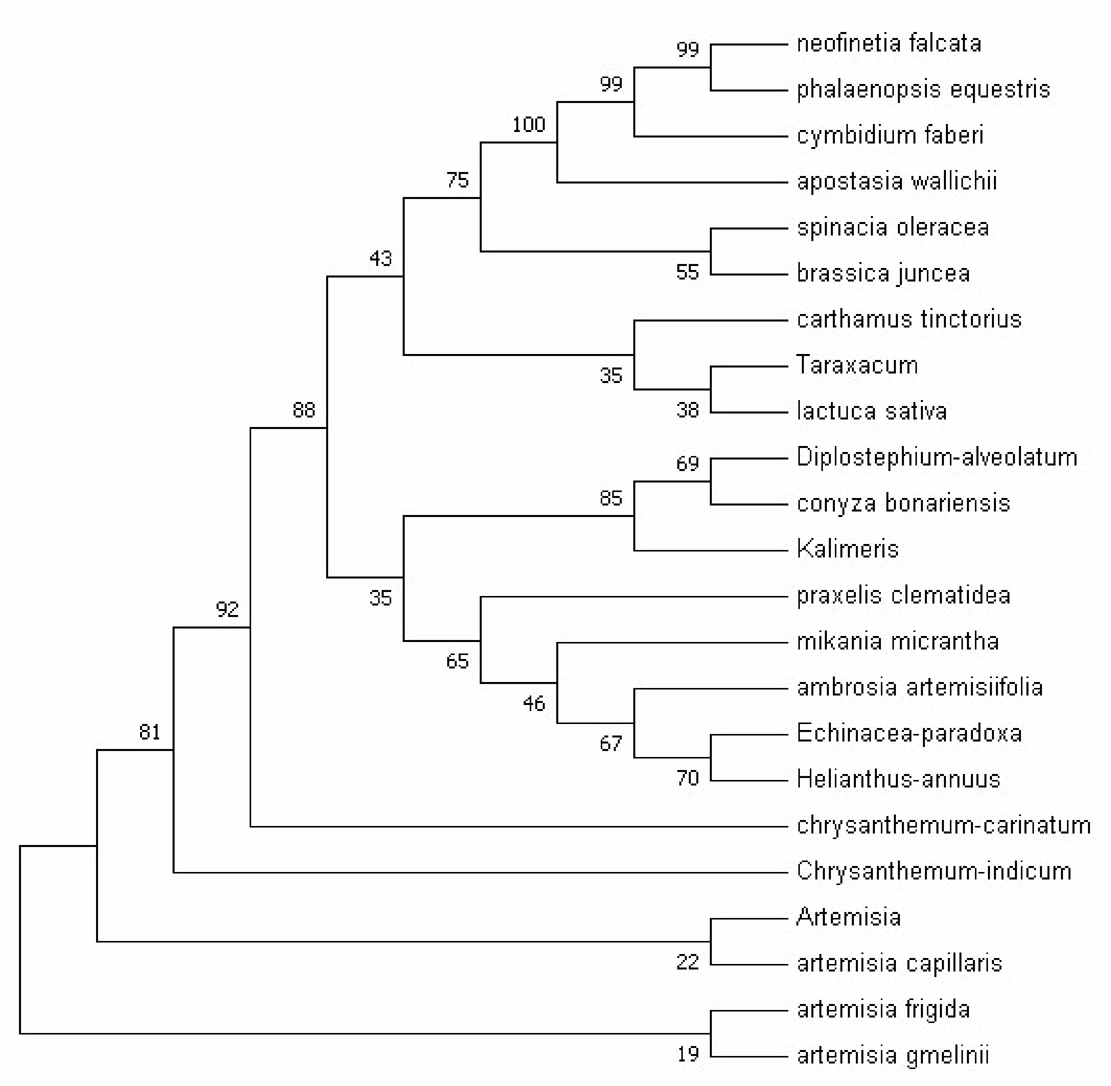

Figure S8. The molecular phylogenetic analysis of the cp protein-coding gene *petD* for 24 samples using the Maximum Likelihood method. The tree was constructed by using MEGA7 . The stability of each tree node was tested by bootstrap analysis with 1000 replicates.

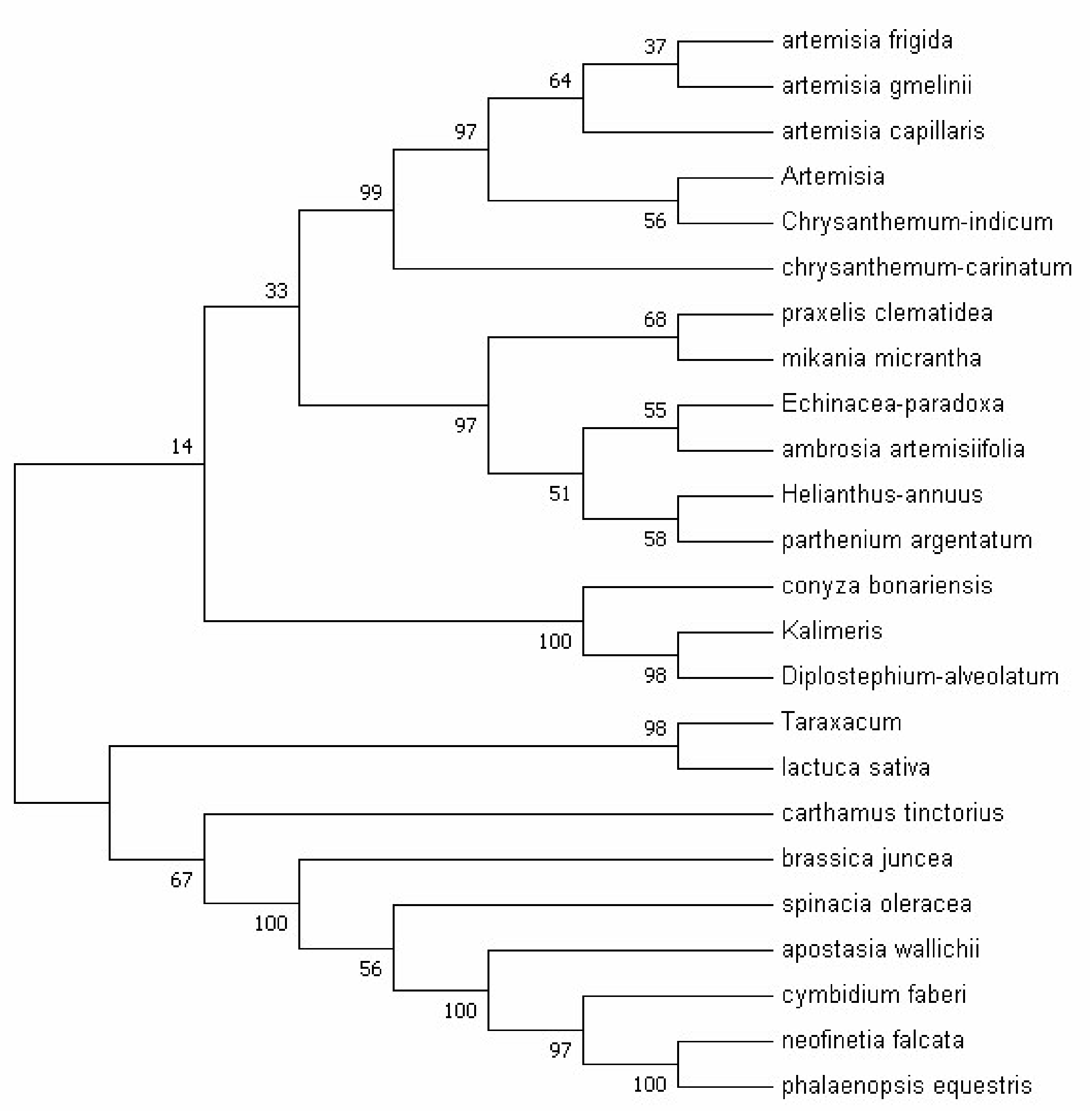

Figure S9. The molecular phylogenetic analysis of the cp protein-coding gene *psaB* for 24 samples using the Maximum Likelihood method. The tree was constructed by using MEGA7 . The stability of each tree node was tested by bootstrap analysis with 1000 replicates.

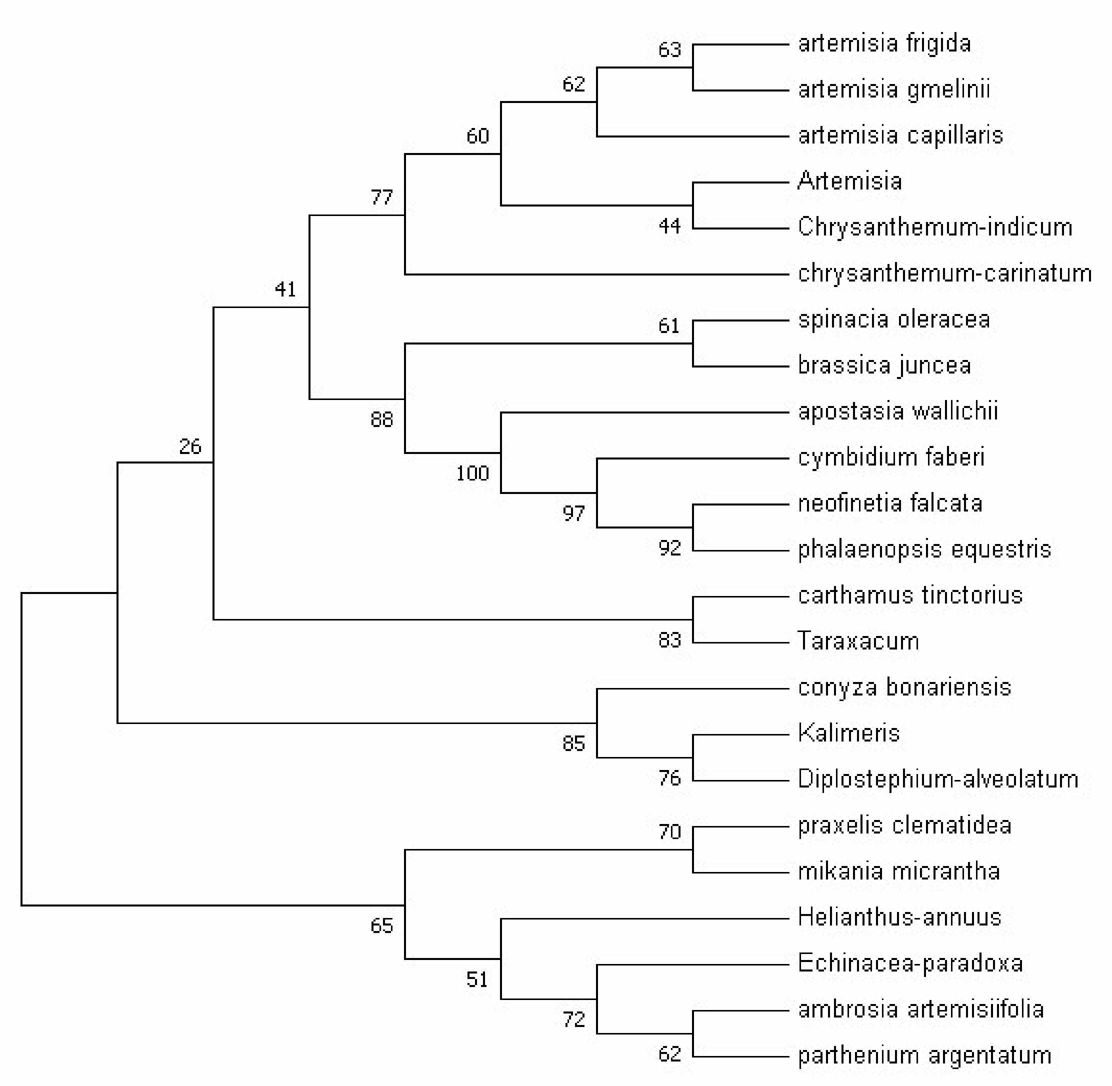

Figure S10. The molecular phylogenetic analysis of the cp protein-coding gene *psbA* for 24 samples using the Maximum Likelihood method. The tree was constructed by using MEGA7 . The stability of each tree node was tested by bootstrap analysis with 1000 replicates.

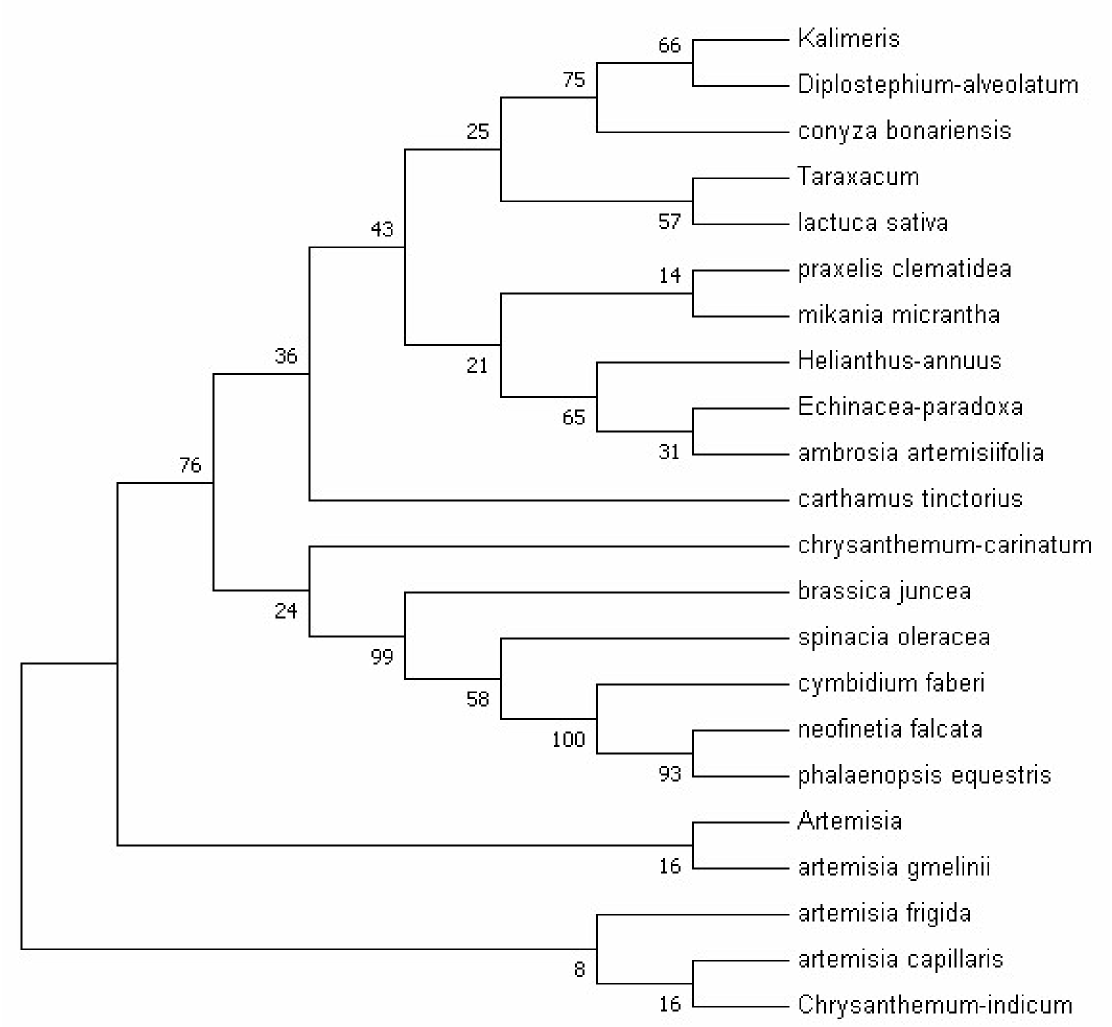

Figure S11. The molecular phylogenetic analysis of the cp protein-coding gene *rpl2* for 24 samples using the Maximum Likelihood method. The tree was constructed by using MEGA7 . The stability of each tree node was tested by bootstrap analysis with 1000 replicates.

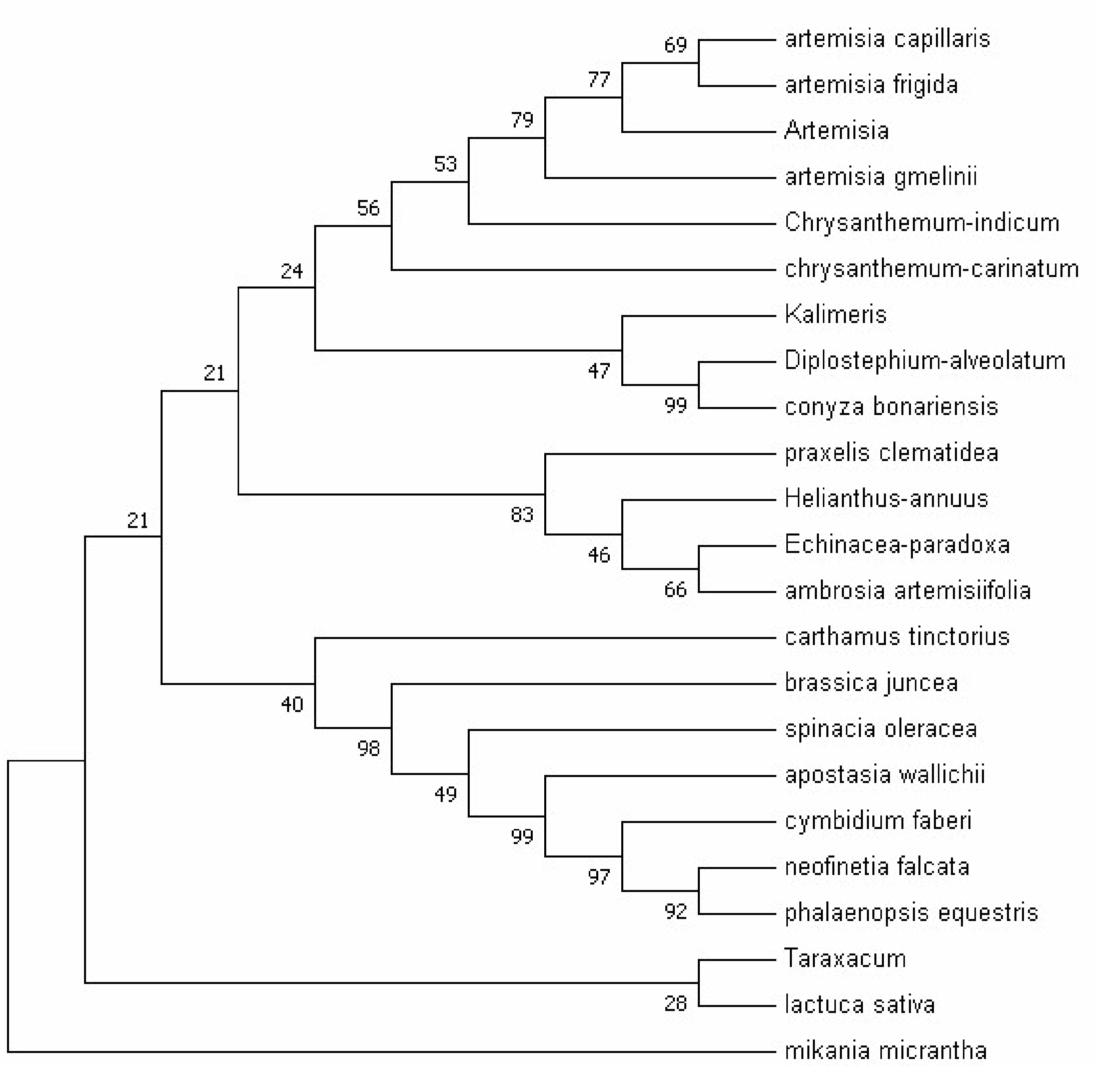

Figure S12. The molecular phylogenetic analysis of the cp protein-coding gene *rpl16* for 24 samples using the Maximum Likelihood method. The tree was constructed by using MEGA7 . The stability of each tree node was tested by bootstrap analysis with 1000 replicates.

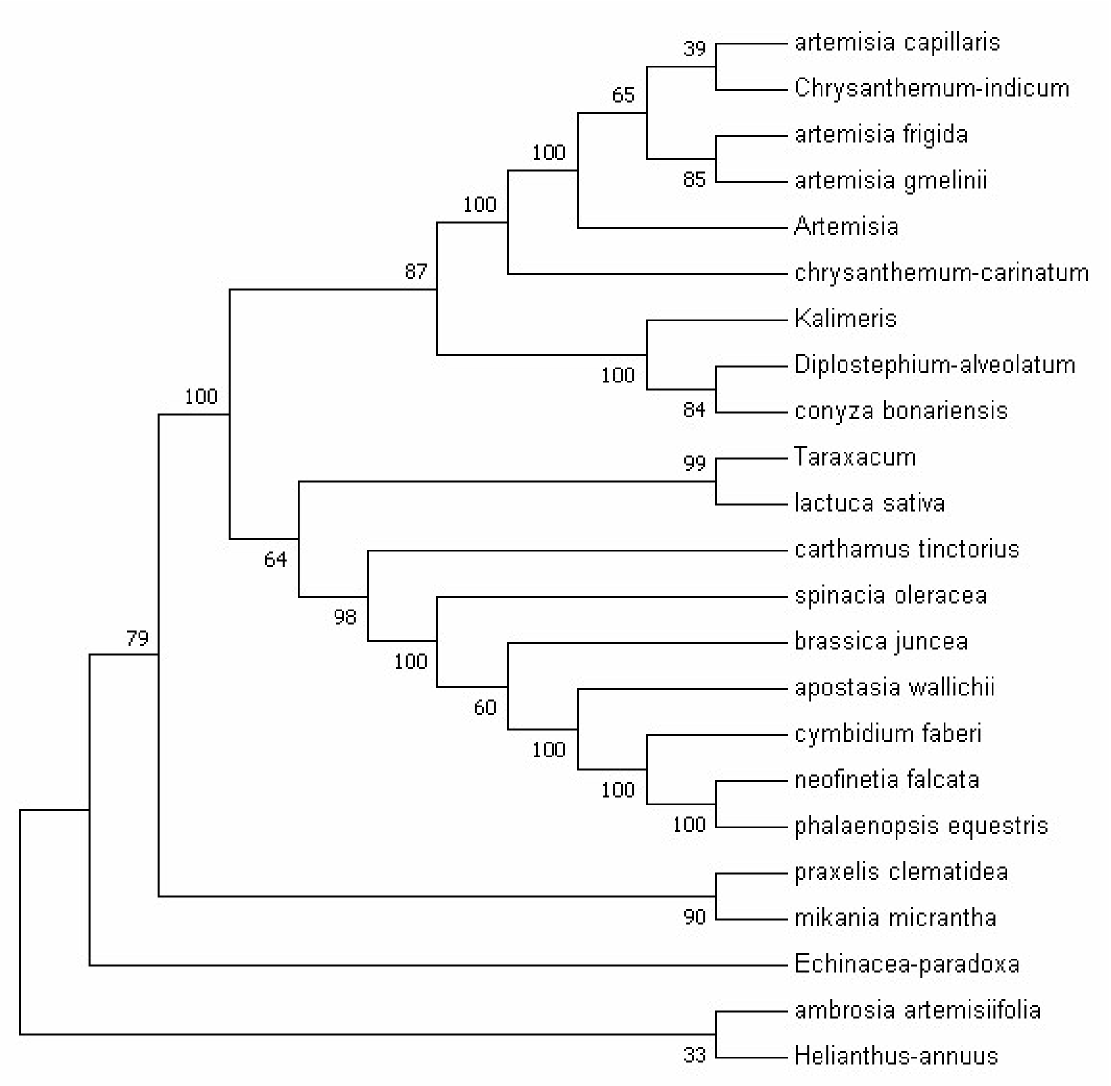

Figure S13. The molecular phylogenetic analysis of the cp protein-coding gene *rpoB* for 24 samples using the Maximum Likelihood method. The tree was constructed by using MEGA7 . The stability of each tree node was tested by bootstrap analysis with 1000 replicates.

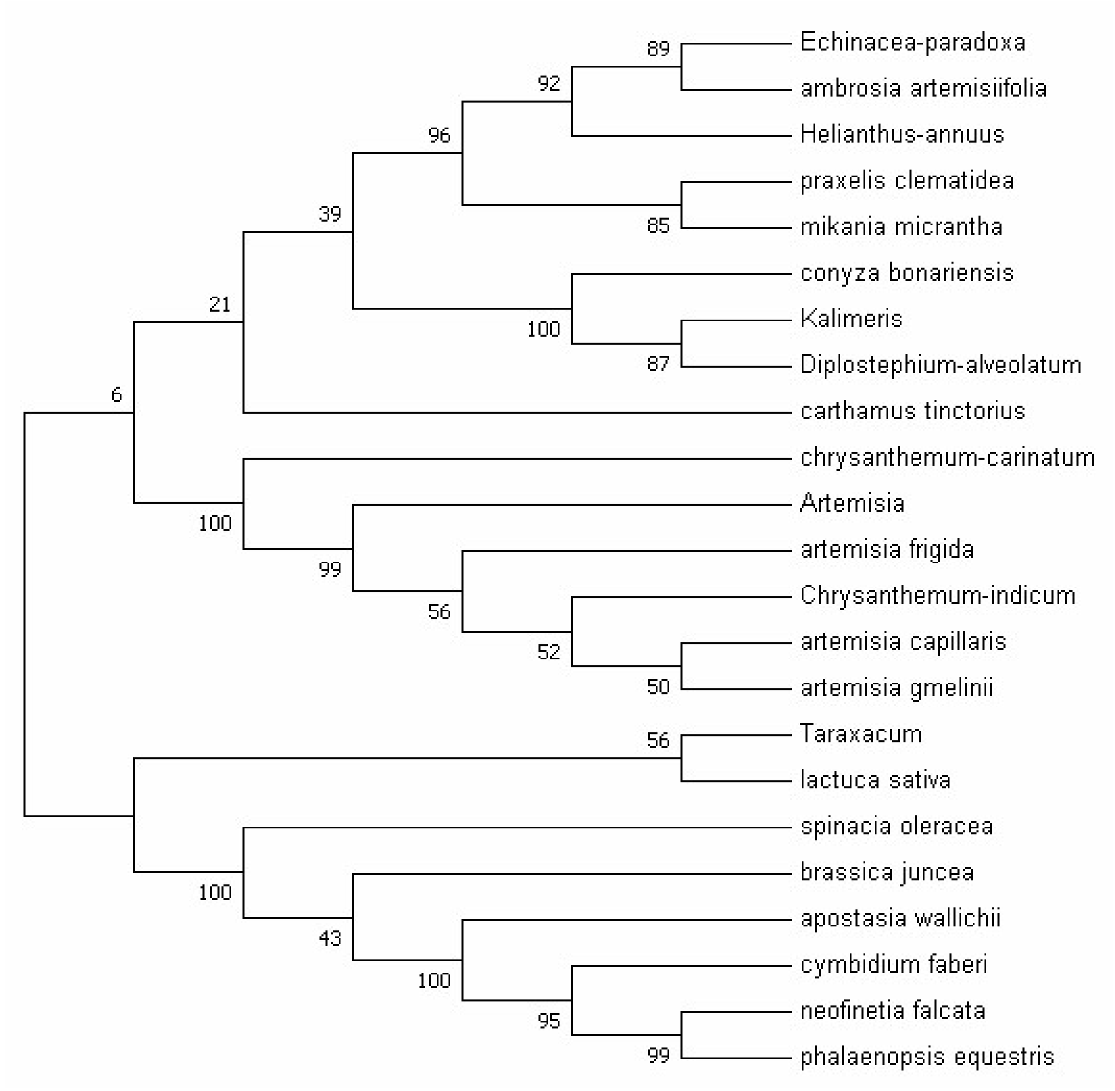

Figure S14. The molecular phylogenetic analysis of the cp protein-coding gene *rpoC1* for 24 samples using the Maximum Likelihood method. The tree was constructed by using MEGA7 . The stability of each tree node was tested by bootstrap analysis with 1000 replicates.

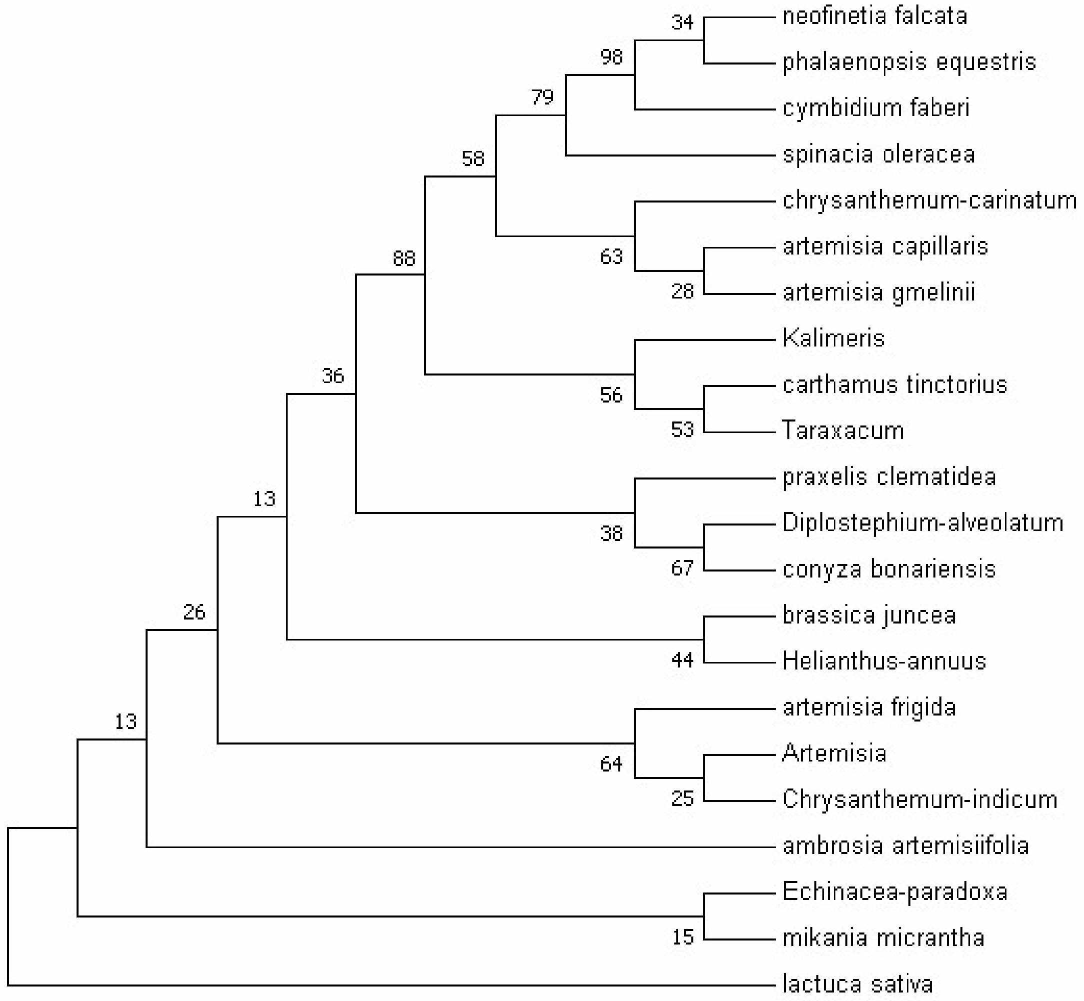

Figure S15. The molecular phylogenetic analysis of the cp protein-coding gene *rps12* for 24 samples using the Maximum Likelihood method. The tree was constructed by using MEGA7 . The stability of each tree node was tested by bootstrap analysis with 1000 replicates.

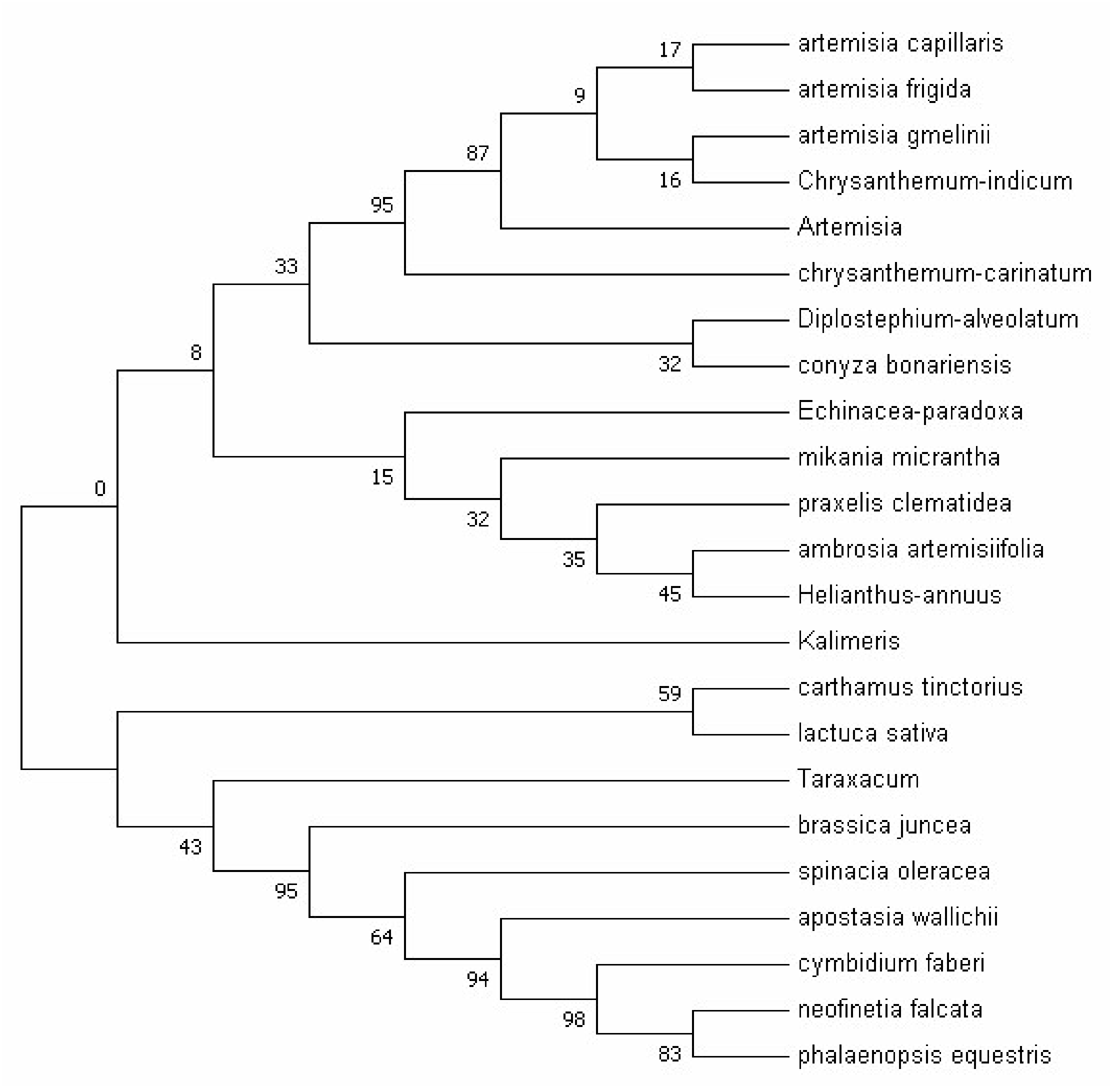

Figure S16. The molecular phylogenetic analysis of the cp protein-coding gene *rps16* for 24 samples using the Maximum Likelihood method. The tree was constructed by using MEGA7 . The stability of each tree node was tested by bootstrap analysis with 1000 replicates.

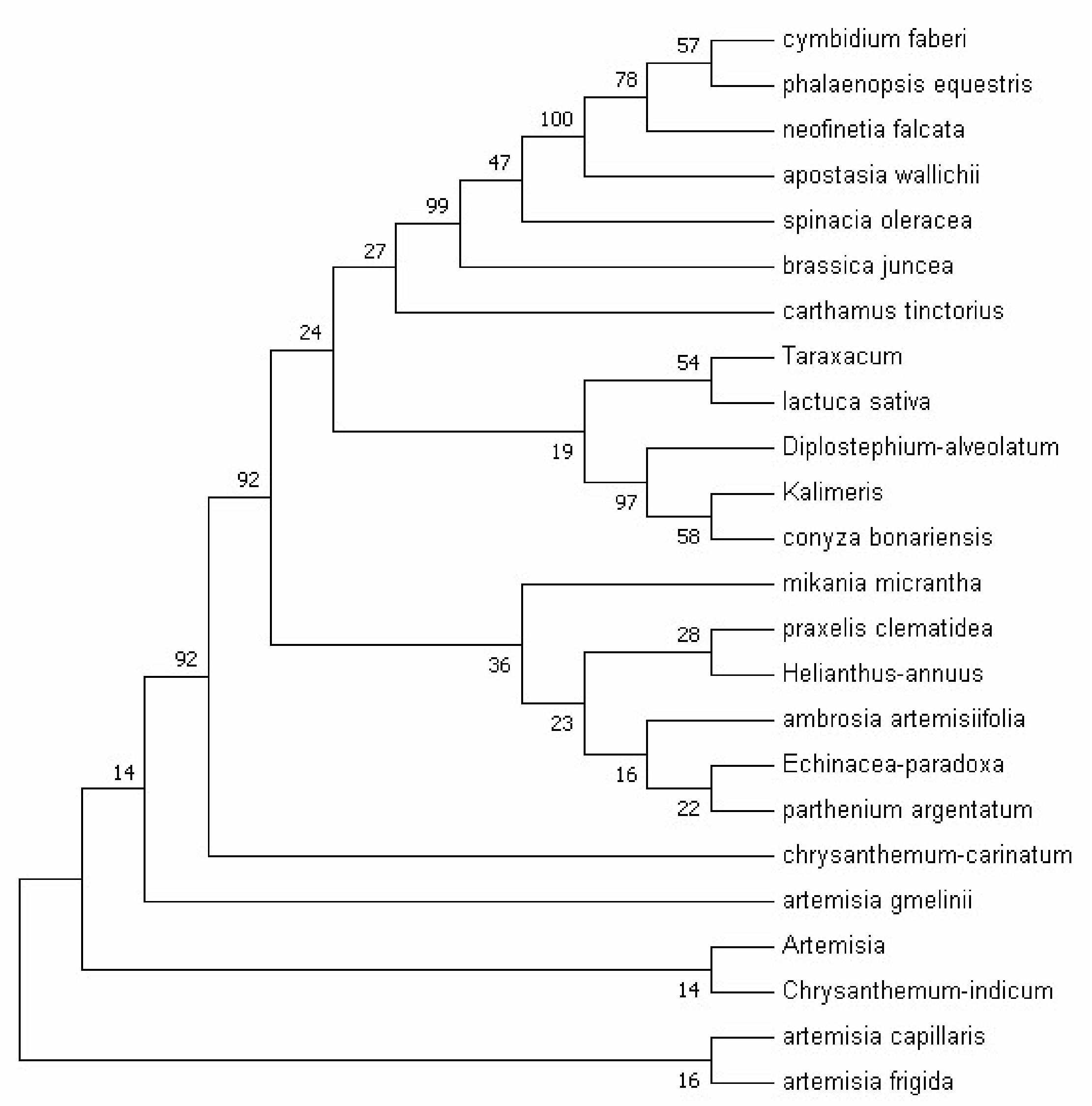

Figure S17. The molecular phylogenetic analysis of the cp protein-coding gene *rps19* for 24 samples using the Maximum Likelihood method. The tree was constructed by using MEGA7 . The stability of each tree node was tested by bootstrap analysis with 1000 replicates.

Table S1. The inversion of the SSC area in different species of *Asteraceae*

Organism Name	Size(Mb)	GC%	Type	Replicons	CDS	Release Date	Inverted
Achyrachaena mollis	0.150468	37.5714	chloroplast	NC_036504.1/MF663009.1	83	2017-12-18T00:00:00Z	Y
Ageratina adenophora	0.150698	37.4617	chloroplast	NC_015621.1/JF826503.1	86	2011-06-02T00:00:00Z	Y
Ambrosia artemisiifolia	0.152215	37.6126	chloroplast	NC_035875.1/MF362689.1	87	2017-09-13T00:00:00Z	Y
Ambrosia trifida	0.15204	37.6217	chloroplast	NC_036810.1/MG029118.1	86	2018-02-06T00:00:00Z	Y
Anaphalis sinica	0.152718	37.1194	chloroplast	NC_034648.1/KX148081.1	85	2017-05-24T00:00:00Z	Y
Archibaccharis asperifolia	0.151984	37.3717	chloroplast	NC_034848.1/KX063859.1	85	2017-06-02T00:00:00Z	Y
Artemisia argyi	0.151192	37.4597	chloroplast	NC_030785.1/KM386991.1	84	2016-08-16T00:00:00Z	N
Artemisia capillaris	0.151056	37.4596	chloroplast	NC_031400.1/KU736963.1	88	2016-10-13T00:00:00Z	N
Artemisia frigida	0.151076	37.4758	chloroplast	NC_020607.1/JX293720.1	87	2013-03-25T00:00:00Z	N
Artemisia gmelinii	0.151318	37.4232	chloroplast	NC_031399.1/KU736962.1	88	2016-10-13T00:00:00Z	N
Artemisia montana	0.15113	37.477	chloroplast	NC_025910.1/KF887960.1	86	2014-12-16T00:00:00Z	N
Aster altaicus	0.152446	37.3424	chloroplast	NC_034996.1/KX352465.1	84	2017-06-13T00:00:00Z	N
Aster spathulifolius	0.14951	37.7085	chloroplast	NC_027434.1/KF279514.1	87	2015-07-14T00:00:00Z	Y
Aztecaster matudae	0.151925	37.4092	chloroplast	NC_034898.1/KX063935.1	85	2017-06-02T00:00:00Z	Y
Baccharis genistelloides	0.153239	37.1668	chloroplast	NC_034852.1/KX063864.1	84	2017-06-02T00:00:00Z	Y
Baccharis tricuneata	0.15236	37.2978	chloroplast	NC_034868.1/KX063888.1	85	2017-06-02T00:00:00Z	Y
Blakiella bartsiifolia	0.151965	37.2875	chloroplast	NC_034866.1/KX063886.1	85	2017-06-02T00:00:00Z	Y
Carthamus tinctorius	0.153114	37.7869	chloroplast	NC_030783.1/KM207677.1	84	2016-08-16T00:00:00Z	N
Centaurea diffusa	0.152559	37.731	chloroplast	NC_024286.1/KJ690264.1	90	2014-06-11T00:00:00Z	N
Conyza bonariensis	0.153014	37.1626	chloroplast	NC_035884.1/MF276802.1	87	2017-09-13T00:00:00Z	Y
Cynara baetica	0.152548	37.7108	chloroplast	NC_028005.1/KP842706.1	87	2015-10-08T00:00:00Z	Y
Cynara cornigera	0.15255	37.7083	chloroplast	NC_028006.1/KP842707.1	87	2015-10-08T00:00:00Z	Y
Cynara humilis	0.152585	37.7049	chloroplast	NC_027113.1/KP299292.1	87	2015-05-14T00:00:00Z	Y

Dendrosenecio battiscombei	0.150556	37.4625	chloroplast	NC_036833.1/KY434195.1	88	2018-02-06T00:00:00Z	Y
Dendrosenecio keniensis	0.150548	37.4704	chloroplast	NC_036832.1/KY434194.1	89	2018-02-06T00:00:00Z	Y
Dendrosenecio keniodendron	0.150555	37.4614	chloroplast	NC_036831.1/KY434193.1	89	2018-02-06T00:00:00Z	Y
Diplostephium alveolatum	0.152265	37.3691	chloroplast	NC_034847.1/KX063856.1	85	2017-06-02T00:00:00Z	Y
Diplostephium antioquense	0.152027	37.3894	chloroplast	NC_034876.1/KX063898.1	85	2017-06-02T00:00:00Z	Y
Diplostephium apiculatum	0.152277	37.3523	chloroplast	NC_034902.1/KX063943.1	85	2017-06-02T00:00:00Z	Y
Diplostephium azureum	0.152124	37.3426	chloroplast	NC_034882.1/KX063907.1	85	2017-06-02T00:00:00Z	Y
Diplostephium barclayanum	0.151974	37.3893	chloroplast	NC_034853.1/KX063865.1	85	2017-06-02T00:00:00Z	Y
Diplostephium cajamarquillense	0.15207	37.3841	chloroplast	NC_034872.1/KX063894.1	85	2017-06-02T00:00:00Z	Y
Diplostephium callilepis	0.15211	37.3749	chloroplast	NC_034856.1/KX063870.1	85	2017-06-02T00:00:00Z	Y
Diplostephium camargoanum	0.152249	37.3585	chloroplast	NC_034897.1/KX063933.1	85	2017-06-02T00:00:00Z	Y
Diplostephium cayambense	0.151206	37.4853	chloroplast	NC_034886.1/KX063912.1	85	2017-06-02T00:00:00Z	Y
Diplostephium cinerascens	0.152169	37.3775	chloroplast	NC_034850.1/KX063862.1	85	2017-06-02T00:00:00Z	Y
Diplostephium cinereum	0.152554	37.314	chloroplast	NC_034869.1/KX063889.1	85	2017-06-02T00:00:00Z	Y
Diplostephium colombianum	0.152314	37.3452	chloroplast	NC_034861.1/KX063876.1	85	2017-06-02T00:00:00Z	Y
Diplostephium coriaceum	0.15229	37.338	chloroplast	NC_034899.1/KX063937.1	85	2017-06-02T00:00:00Z	Y
Diplostephium costaricense	0.152048	37.4092	chloroplast	NC_034879.1/KX063901.1	85	2017-06-02T00:00:00Z	Y
Diplostephium crypteriophyllum	0.151665	37.3943	chloroplast	NC_034881.1/KX063905.1	85	2017-06-02T00:00:00Z	Y
Diplostephium empetrifoli um	0.152461	37.3466	chloroplast	NC_034891.1/KX063925.1	85	2017-06-02T00:00:00Z	Y
Diplostephium ericoides	0.151982	37.3992	chloroplast	NC_034870.1/KX063892.1	85	2017-06-02T00:00:00Z	Y
Diplostephium eriophorum	0.151802	37.4317	chloroplast	NC_034874.1/KX063896.1	85	2017-06-02T00:00:00Z	Y
Diplostephium espinosae	0.152091	37.3592	chloroplast	NC_034880.1/KX063903.1	85	2017-06-02T00:00:00Z	Y
Diplostephium floribundum	0.151768	37.4163	chloroplast	NC_034857.1/KX063872.1	85	2017-06-02T00:00:00Z	Y
Diplostephium foliosissimum	0.15226	37.325	chloroplast	NC_034883.1/KX063909.1	85	2017-06-02T00:00:00Z	Y
Diplostephium frontinense	0.152365	37.3314	chloroplast	NC_034893.1/KX063927.1	85	2017-06-02T00:00:00Z	Y

Diplostephium glandulosum	0.1514	37.4736	chloroplast	NC_034854.1/KX063866.1	85	2017-06-02T00:00:00Z	Y
Diplostephium glutinosum	0.152229	37.3359	chloroplast	NC_034875.1/KX063897.1	85	2017-06-02T00:00:00Z	Y
Diplostephium gnidioides	0.152144	37.3692	chloroplast	NC_034867.1/KX063887.1	85	2017-06-02T00:00:00Z	Y
Diplostephium goodspeedii	0.152006	37.3939	chloroplast	NC_034901.1/KX063940.1	85	2017-06-02T00:00:00Z	Y
Diplostephium gynoxyoides	0.151662	37.4497	chloroplast	NC_034862.1/KX063877.1	85	2017-06-02T00:00:00Z	Y
Diplostephium haenkei	0.152292	37.3329	chloroplast	NC_034871.1/KX063893.1	85	2017-06-02T00:00:00Z	Y
Diplostephium hartwegii	0.151994	37.387	chloroplast	NC_034832.1/KX063880.1	85	2017-05-26T00:00:00Z	Y
Diplostephium heterophyllum	0.152223	37.3695	chloroplast	NC_034896.1/KX063931.1	85	2017-06-02T00:00:00Z	Y
Diplostephium hippophae	0.152197	37.3562	chloroplast	NC_034831.1/KX063944.1	85	2017-05-26T00:00:00Z	Y
Diplostephium huertasii	0.151684	37.4232	chloroplast	NC_034812.1/KX063915.1	85	2017-05-26T00:00:00Z	Y
Diplostephium inesianum	0.152328	37.3431	chloroplast	NC_034895.1/KX063930.1	85	2017-06-02T00:00:00Z	Y
Diplostephium jaramilloi	0.151879	37.4311	chloroplast	NC_034894.1/KX063928.1	85	2017-06-02T00:00:00Z	Y
Diplostephium jelskii	0.152142	37.3763	chloroplast	NC_034811.1/KX063860.1	85	2017-05-26T00:00:00Z	Y
Diplostephium jenesanum	0.150924	37.5162	chloroplast	NC_034829.1/KX063934.1	84	2017-05-26T00:00:00Z	Y
Diplostephium juajibioyi	0.152282	37.3518	chloroplast	NC_034822.1/KX063913.1	85	2017-05-26T00:00:00Z	Y
Diplostephium juniperinum	0.152454	37.3398	chloroplast	NC_034813.1/KX063883.1	85	2017-05-26T00:00:00Z	Y
Diplostephium lacunosum	0.152247	37.3636	chloroplast	NC_034878.1/KX063900.1	85	2017-06-02T00:00:00Z	Y
Diplostephium lechleri	0.152067	37.3743	chloroplast	NC_034816.1/KX063868.1	85	2017-05-26T00:00:00Z	Y
Diplostephium meyenii	0.152537	37.3188	chloroplast	NC_034824.1/KX063919.1	85	2017-05-26T00:00:00Z	Y
Diplostephium mutiscuanum	0.152282	37.3583	chloroplast	NC_034827.1/KX063929.1	85	2017-05-26T00:00:00Z	Y
Diplostephium oblanceolatum	0.151895	37.412	chloroplast	NC_034830.1/KX063941.1	85	2017-05-26T00:00:00Z	Y
Diplostephium oblongifolium	0.152282	37.3557	chloroplast	NC_034818.1/KX063906.1	85	2017-05-26T00:00:00Z	Y
Diplostephium obtusum	0.152179	37.3698	chloroplast	NC_034814.1/KX063920.1	85	2017-05-26T00:00:00Z	Y
Diplostephium ochraceum	0.151555	37.4524	chloroplast	NC_034903.1/KX063945.1	85	2017-06-02T00:00:00Z	Y
Diplostephium oxapampanum	*0.151637*	37.4295	chloroplast	NC_034815.1/KX063884.1	85	2017-05-26T00:00:00Z	Y

Diplostephium phylicoides	*0.152172*	37.3794	chloroplast	NC_034873.1/KX063895.1	85	2017-06-02T00:00:00Z	Y
Diplostephium pulchrum	*0.152236*	37.346	chloroplast	NC_034810.1/KX063857.1	85	2017-05-26T00:00:00Z	Y
Diplostephium revolutum	*0.151823*	37.3995	chloroplast	NC_034863.1/KX063878.1	85	2017-06-02T00:00:00Z	Y
Diplostephium rhododendroides	*0.152289*	37.35	chloroplast	NC_034820.1/KX063885.1	85	2017-05-26T00:00:00Z	Y
Diplostephium rhomboidale	*0.152186*	37.3635	chloroplast	NC_034859.1/KX063874.1	85	2017-06-02T00:00:00Z	Y
Diplostephium romeroi	*0.152275*	37.3482	chloroplast	NC_034885.1/KX063911.1	85	2017-06-02T00:00:00Z	Y
Diplostephium rosmarinifolium	*0.152216*	37.364	chloroplast	NC_034900.1/KX063939.1	85	2017-06-02T00:00:00Z	Y
Diplostephium rupestre	*0.152027*	37.3447	chloroplast	NC_034865.1/KX063882.1	85	2017-06-02T00:00:00Z	Y
Diplostephium sagasteguii	*0.152214*	37.3514	chloroplast	NC_034828.1/KX063932.1	85	2017-05-26T00:00:00Z	Y
Diplostephium schultzii	*0.152252*	37.3683	chloroplast	NC_034892.1/KX063926.1	85	2017-06-02T00:00:00Z	Y
Diplostephium serratifolium	*0.15206*	37.3885	chloroplast	NC_034826.1/KX063924.1	85	2017-05-26T00:00:00Z	Y
Diplostephium spinulosum	*0.152155*	37.3849	chloroplast	NC_034823.1/KX063917.1	85	2017-05-26T00:00:00Z	Y
Diplostephium tachirense	*0.152197*	37.3621	chloroplast	NC_034825.1/KX063922.1	85	2017-05-26T00:00:00Z	Y
Diplostephium tenuifolium	*0.151911*	37.4252	chloroplast	NC_034860.1/KX063875.1	85	2017-06-02T00:00:00Z	Y
Diplostephium venezuelense	*0.152173*	37.3746	chloroplast	NC_034887.1/KX063914.1	85	2017-06-02T00:00:00Z	Y
Diplostephium violaceum	*0.151387*	37.4946	chloroplast	NC_034817.1/KX063891.1	85	2017-05-26T00:00:00Z	Y
Eclipta prostrata	*0.151757*	37.4849	chloroplast	NC_030773.1/KU361242.1	87	2016-08-16T00:00:00Z	N
Exostigma notobellidiastrum	*0.151699*	37.3285	chloroplast	NC_034864.1/KX063881.1	85	2017-06-02T00:00:00Z	Y
Floscaldasia hypsophila	*0.1522*	37.3167	chloroplast	NC_034888.1/KX063916.1	85	2017-06-02T00:00:00Z	Y
Galinsoga quadriradiata	*0.151917*	37.6554	chloroplast	NC_031853.1/KX752097.1	87	2016-12-05T00:00:00Z	Y
Guizotia abyssinica	*0.151762*	37.6227	chloroplast	NC_010601.1/EU549769.1	85	2008-04-15T00:00:00Z	Y
Helianthus annuus	*0.151104*	37.6198	chloroplast	NC_007977.1/DQ383815.1	85	2006-05-03T00:00:00Z	Y
Helianthus argophyllus	*0.151069*	37.6259	chloroplast	NC_030275.1/KU314500.1	82	2016-05-31T00:00:00Z	Y
Helianthus debilis	*0.151117*	37.6106	chloroplast	NC_030173.1/KU312928.1	85	2016-05-19T00:00:00Z	Y
Heterothalamus alienus	0.152252	37.3565	chloroplast	NC_034855.1/KX063869.1	85	2017-06-02T00:00:00Z	Y

Hinterhubera ericoides	0.150711	37.3277	chloroplast	NC_034884.1/KX063910.1	85	2017-06-02T00:00:00Z	Y
Jacobaea vulgari	0.150689	37.3199	chloroplast	NC_015543.1/HQ234669.1	87	2011-06-02T00:00:00Z	Y
Lactuca sativa	0.152765	37.5492	chloroplast	NC_007578.1/	84	2005-11-08T00:00:00Z	N
Laennecia sophiifolia	0.151899	37.3452	chloroplast	NC_034877.1/KX063899.1	85	2017-06-02T00:00:00Z	Y
Laestadia muscicola	0.152366	37.2426	chloroplast	NC_034858.1/KX063873.1	85	2017-06-02T00:00:00Z	Y
Lagenophora cuchumatanica	0.152462	37.2486	chloroplast	NC_034819.1/KX063879.1	85	2017-05-26T00:00:00Z	Y
Leontopodium leiolepis	0.151072	37.3213	chloroplast	NC_027835.1/KM267636.1	85	2015-09-14T00:00:00Z	Y
Llerasia caucana	0.152015	37.491	chloroplast	NC_034821.1/KX063908.1	85	2017-05-26T00:00:00Z	Y
Mikania micrantha	0.152092	37.5799	chloroplast	NC_031833.1/KX154571.1	86	2016-11-14T00:00:00Z	Y
Oritrophium peruvianum	0.151723	37.3576	chloroplast	NC_034849.1/KX063861.1	85	2017-06-02T00:00:00Z	Y
Parastrephia quadrangularis	0.152427	37.3084	chloroplast	NC_034890.1/KX063923.1	85	2017-06-02T00:00:00Z	Y
Parthenium argentatum	0.152803	37.6079	chloroplast	NC_013553.1/GU120098.1	55	2009-12-15T00:00:00Z	Y
Pericallis hybrida	0.151267	37.3261	chloroplast	NC_031898.1/KT285537.1	87	2016-11-14T00:00:00Z	Y
Praxelis clematidea	0.15141	37.2333	chloroplast	NC_023833.1/KF922320.1	84	2014-03-26T00:00:00Z	Y
Saussurea chabyoungsanica	0.152446	37.6651	chloroplast	NC_036677.1/KX622799.1	87	2018-01-18T00:00:00Z	N
Saussurea involucrata	0.15249	37.6707	chloroplast	NC_029465.1/KU041648.1	90	2016-03-01T00:00:00Z	N
Saussurea polylepis	0.152488	37.6594	chloroplast	NC_036490.1/MF695711.1	87	2017-12-18T00:00:00Z	N
Silybum marianum	0.153202	37.7378	chloroplast	NC_028027.1/KT267161.1	87	2015-10-08T00:00:00Z	Y
Soliva sessilis	0.150784	37.4649	chloroplast	NC_034851.1/KX063863.1	85	2017-06-02T00:00:00Z	Y
Taraxacum amplum	0.151349	37.6791	chloroplast	NC_031816.1/KX499525.1	86	2016-11-14T00:00:00Z	Y
Taraxacum brevicorniculatum	0.151282	37.6786	chloroplast	NC_032056.1/KX198559.1	82	2016-12-07T00:00:00Z	Y
Taraxacum kok-saghyz	0.151338	37.7083	chloroplast	NC_032057.1/KX198560.1	82	2016-12-07T00:00:00Z	Y
Taraxacum mongolicum	0.151451	37.6676	chloroplast	NC_031396.1/KU736961.1	87	2016-10-13T00:00:00Z	N
Taraxacum obtusifrons	0.151322	37.6759	chloroplast	NC_031815.1/KX499524.1	85	2016-11-14T00:00:00Z	Y
Taraxacum officinale	0.151324	37.686	chloroplast	NC_030772.1/KU361241.1	87	2016-08-16T00:00:00Z	N

Taraxacum platycarpum	0.151307	37.6903	chloroplast	NC_031395.1/KU736960.1	87	2016-10-13T00:00:00Z	N
Westoniella kohkemperi	0.151824	37.3571	chloroplast	NC_034889.1/KX063921.1	85	2017-06-02T00:00:00Z	Y
Chrysanthemum indicum	0.150972	37.4785	chloroplast	NC_020320.1/JN867589.1	83	2013-02-25T00:00:00Z	Y
Chrysanthemum x morifolium	0.151033	37.4806	chloroplast	NC_020092.1/JQ362483.1	85	2013-01-10T00:00:00Z	Y
Chrysanthemum carinatum Schousb	0.149752	37.5	chloroplast	MG710386	85		Y
Kalimeris indica	0.152885	37.5	chloroplast	MG710387	85		Y
